# Topic analysis on publications and patents toward fully automated translational science benefits model impact extraction

**DOI:** 10.3389/frma.2025.1596687

**Published:** 2025-09-23

**Authors:** Tejaswini Manjunath, Eline Appelmans, Sinem Balta, Dominick DiMercurio, Claudia Avalos, Karen Stark

**Affiliations:** Digital Infuzion LLC, Rockville, MD, United States

**Keywords:** Translational Science Benefits Model (TSBM), Natural Language Processing (NLP), Latent Dirichlet Allocation (LDA), Clinical and Translational Science Award (CTSA), impact analysis, Artificial Intelligence (AI), Large Language Model (LLM), topic analysis

## Abstract

**Background:**

The Clinical and Translational Science Award (CTSA) program, funded by the National Center for Advancing Translational Sciences (NCATS), has supported over 65 hubs, generating 118,490 publications from 2006 to 2021. Measuring the impact of these outputs remains challenging, as traditional bibliometric methods fail to capture patents, policy contributions, and clinical implementation. The Translational Science Benefits Model (TSBM) provides a structured framework for assessing clinical, community, economic, and policy benefits, but its manual application is resource-intensive. Advances in Natural Language Processing (NLP) and Artificial Intelligence (AI) offer a scalable solution for automating benefit extraction from large research datasets.

**Objective:**

This study presents an NLP-driven pipeline that automates the extraction of TSBM benefits from research outputs using Latent Dirichlet Allocation (LDA) topic modeling to enable efficient, scalable, and reproducible impact analysis. The application of NLP allows the discovery of topics and benefits to emerge from the very large corpus of CTSA documents without requiring directed searches or preconceived benefits for data mining.

**Methods:**

We applied LDA topic modeling to publications, patents, and grants and mapped the topics to TSBM benefits using subject matter expert (SME) validation. Impact visualizations, including heatmaps and t-SNE plots, highlighted benefit distributions across the corpus and CTSA hubs.

**Results:**

Spanning CTSA hub grants awarded from 2006 to 2023, our analysis corpus comprised 1,296 projects, 127,958 publications and 352 patents. Applying our NLP-driven pipeline to deduplicated data, we found that clinical and community benefits were the most frequently extracted benefits from publications and projects, reflecting the patient-centered and community-driven nature of CTSA research. Economic and policy benefits were less frequently identified, prompting the inclusion of patent data to better capture commercialization impacts. The Publications LDA Model proved the most effective for benefit extraction for publications and projects. All patents were automatically tagged as economic benefits, given their intrinsic focus on commercialization and in accordance with TSBM guidelines.

**Conclusion:**

Automated NLP-driven benefit extraction enabled a data-driven approach to applying the TSBM at the scale of the entire CTSA program outputs.

## 1 Introduction

Translational science plays a critical role in bridging the gap between scientific discoveries and real-world health outcomes. The National Center for Accelerating Translational Science (NCATS) Clinical and Translational Science Award (CTSA) program has funded more than 65 hubs at leading medical institutions nationwide (National Center for Advancing Translational Sciences, 2024). Between 2006 to 2021 CTSA hubs produced 118,490 publications with 13% of CTSA-supported articles were referenced in policy documents, demonstrating a pivotal role in translating basic research into clinical applications (Llewellyn et al., 2023).

However, measuring the CTSA program's real-world impact remains a challenge. The program's evolving and growing scope and the variety of outputs (from publications, to patents, policy and clinical guidelines) make it difficult to select and deploy methods that are both comprehensive and practical. Traditional bibliometrics reliably quantify publications and citations but systematically miss non-publication contributions, such as policy briefs and clinical implementation guides (Llewellyn et al., 2023), and they offer little insight into the downstream benefits that inform strategic decision-making. Although recent bibliometric innovations have begun to trace connections between research and policy outcomes (Llewellyn et al., 2023), these methods still rely on labor-intensive workflows. As a result, there is a critical need for automated, reproducible approaches that can capture the full spectrum of translational benefits and deliver actionable insights at program scale across all output types.

Historically, some other methods have been applied. The Common Metrics Initiative was originally developed to assess the efficiency of clinical research processes and was used by the CTSAs program to leverage change based on objective results (Daudelin et al., 2020). However, implementing the Common Metrics across the CTSA Program proved to be effort-intensive (Welch et al., 2021). In 2022, the CTSA hubs were tasked with continuing quality improvement programs to make individualized decisions that improve their processes.

In response to the need for a more specific understanding of the impacts of the CTSA research, the Institute for Clinical and Translational Sciences Tracking and Evaluation Team at Washington University in St. Louis developed the Translational Science Benefits Model (TSBM) in 2018 (Luke et al., 2018). The TSBM moves beyond traditional bibliometrics as a sole measure of scientific productivity by offering a framework for assessing the real-world impact of translational research across four domains: clinical, community, economic, and policy benefits.

While the TSBM framework offers a structured approach to capturing the diverse benefits of translational science, even creating a single case study using the model is a labor intensive process with one published case studying taking up to 9 hours to complete, which aligned with our experience also (Swanson et al., 2025). Applying TSBM to the vast corpus of all CTSA program outputs would require infeasibly high levels of subject matter expertise to perform case studies. This manually intensive process limits the scalability of the model for application to the CTSA program as a whole. Given that the CTSA publications contain valuable evidence of benefits, a more efficient solution is needed to highlight directly the many benefits that have been derived from the research funding.

Advances in Natural Language Processing (NLP) and Artificial Intelligence (AI) methods provide capabilities for extracting desired content from such large-scale document corpora. Toolkits such as the Python Natural Language Toolkit (Loper and Bird, 2002) have become commonplace and Large Language Models (LLMs) such as GPT4.0 (OpenAI et al., 2024) provide advanced text understanding and generation.

In addition to saving effort, a key motivation for a data-driven pipeline is that NLP enables benefits and topics to arise from the documents themselves and can extract benefits that may not be evident without such a bottom-up approach. Topic Analysis, such as Latent Dirichlet Allocation (LDA) (Blei et al., 2003), provides a broadly accepted method for analyzing a large data corpus. Without such an NLP system in place, demonstration of value relies largely on “top-down” methods for searching, querying, or targeted data mining that typically require specific pre-conceived ideas around potential benefits.

The Impact Analysis Team of the Coordination, Communication and Operations Support (CCOS) Center for the CTSA program has therefore developed an NLP-based software pipeline to apply the TSBM to the CTSA documents. This pipeline enables rapid identification of potential TSBM benefits across thousands of documents, significantly reducing the manual effort required for such analysis. LDA is applied to pre-processed publication, patent, and grant data to uncover emerging themes and patterns across these datasets. The identified topics are then mapped to TSBM benefits, allowing us to systematically link research outputs to potential impacts. This data-driven approach allows for the emergence of previously unrecognized benefits directly from the text of the program outputs, helps reveal the underlying structure of the data, and enhances our understanding of how different research efforts contribute to translational science benefits.

## 2 Materials and methods

### 2.1 Overview

This paper presents an innovative approach leveraging NLP and AI to automate the extraction of TSBM benefits from unstructured text in publicly available research outputs. By applying advanced topic modeling techniques, we systematically enrich publications, patents, and other research outputs with TSBM benefit categories.

The foundation of this NLP-driven approach is LDA, a generative probabilistic model that identifies latent topics within a collection of text documents by analyzing word distributions and co-localization patterns, where certain words tend to co-occur in similar contexts.

**Figure 1:**
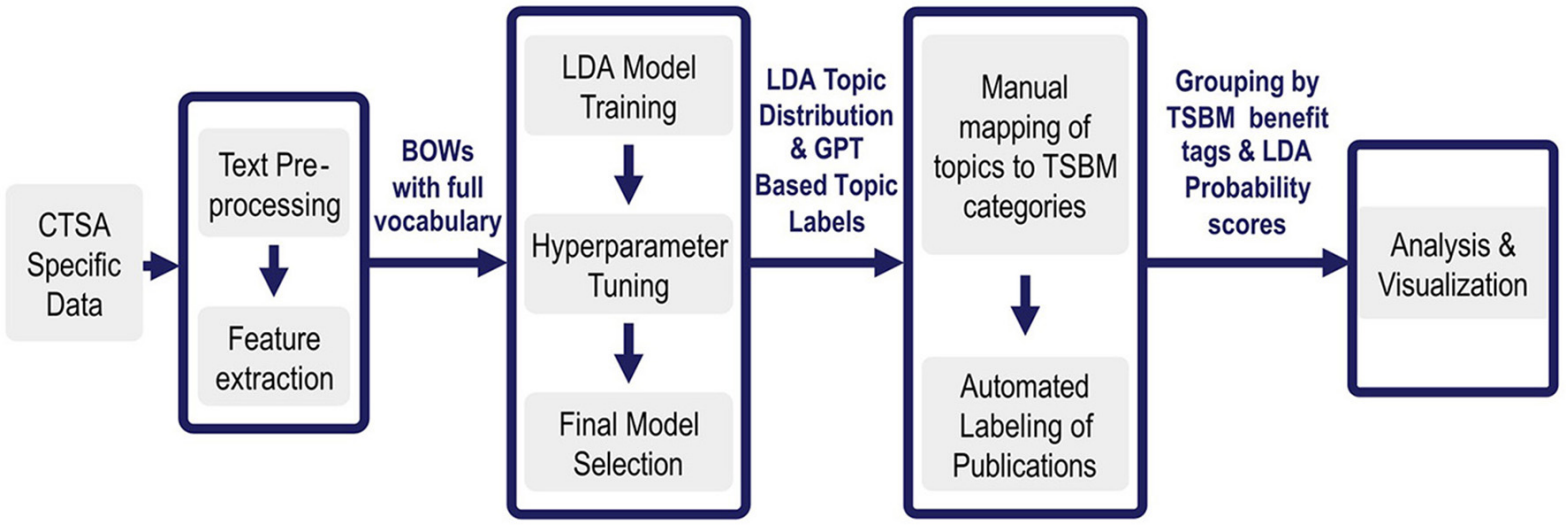
Overview of benefits extraction pipeline. Step 1: The pipeline ingests tens of thousands of CTSA specific documents and pre-processes them into a Bag of Words. Step 2: LDA then extracts a few hundred topics that are most prevalent in them. Step 3: Each topic is then assigned a label by GPT4.0 and given benefits tags by an SME. The appropriate benefits and goal tags are then automatically assigned to each appropriate document based on its prominent topics. Step 4: This enriched set of documents enables further automated analysis and visualizations. For the first time, the tagged datasets enable comprehensive impact analysis and interactive visualizations across over 100,000 documents, providing NCATS with a first-ever large-scale analysis of CTSA research benefits.

An overview of the NLP pipeline methods is given in Figure 1. In Step 1, CTSA specific data (National Center for Advancing Translational Sciences, 2024) is pre-processed and prepared for input to the LDA algorithm. In Step 2, The LDA is tuned to optimize its performance on the specific corpus of data. After LDA has created topics from the data, it assigns each document its probability of discussing each specific topic. Next, we use GPT4.0, an LLM, to assign short descriptive titles to each topic. Since this occurs on a manageable set of no more than a few hundred topics rather than tens of thousands of documents, it is feasible to validate the labels using a subject matter expert (SME). The LLM is thus used as an enhancement for understanding the topics, rather than as a foundational tool for generating them.

During expert validation in Step 3, each topic is manually assigned TSBM benefit tags based on the most important words in each topic, as described in detail in Step 3 below. Once this is completed for each topic, the automation then takes each document, looks at the probability scores assigned by LDA for each of its topics, and tags each document in turn with all the appropriate TSBM benefit tags based on the tags assigned to each of its high probability topics. This means that once a topic is tagged with TSBM benefits, all documents having a high probability for that topic inherit the same benefit tags. For example, if Topic 5 is linked to a community benefit, then all documents with a high probability score for Topic 5 receive the community benefit tag, ensuring consistent and scalable classification.

Finally, in Step 4, we conduct multiple types of benefits analysis and produce visualizations based on the topic-enriched and tagged data sets. The details for each of these major steps are presented next, followed by a discussion of the data sources and the flow of data through the pipeline.

### 2.2 Detailed methods

#### 2.2.1 Step 1 text pre-processing

The first step required to apply LDA is text pre-processing. Its primary objective is to standardize the text representation while minimizing irrelevant or noisy information and we used well-established NLP pre-processing techniques to prepare the data for analysis, primarily using the Python NLTK (Loper and Bird, 2002):

- Convert all text to lowercase to avoid duplication of terms due to differential capitalization
- Remove special characters while retaining hyphenation that enhances meaning
- Tokenization to break text into words
- Part-of-speech tagging so that the same root word is counted separately for verb or noun forms
- Lemmatization (using WordNet) so that all forms of the same root word are counted together
- Remove standard stop words (such as “the” and “a”) that convey little meaning
- Removal of custom stop words including common acronyms, organizational names, and other irrelevant terms empirically derived from the CTSA corpus
- Convert numerical values (except dates) to words so all forms are commonly identified

LDA requires a standard Bag of Words (BoW) (Qader et al., 2019) representation that contains the features (words) to be used as input. We used CountVectorizer from SciKit-learn with the following parameters to create the BoW:

- max_df = 1.0: Includes all words regardless of their frequency.
- min_df = 2: Excludes terms that appear in fewer than two documents.
- n-gram_range = (1, 3): Captures unigrams, bigrams, and trigrams.

We chose CountVectorizer over TfidfVectorizer based on established best practices for LDA pre-processing. LDA is a generative probabilistic model that assumes documents are generated from mixtures of topics, where topics are distributions over words. The model works with raw word counts (document-term frequency) rather than normalized weights, as it needs the actual frequency information to estimate the multinomial distributions that underlie the topic-word and document-topic relationships.

TfidfVectorizer applies term frequency-inverse document frequency weighting, which downweights common words across the corpus. However, this pre-processing can interfere with LDA's ability to identify topics, as some frequently occurring words may actually be important topic indicators when considered in their proper distributional context. Additionally, LDA's internal processes already account for word frequency patterns through its Dirichlet priors and sampling procedures.

This choice aligns with standard LDA implementations and recommendations in the literature (Blei et al., 2003). Scikit-learn was selected for its robust, well-documented implementation and seamless integration with our pre-processing pipeline.

These pre-processing steps were applied separately to each of the datasets used, and the BoW for each dataset was given as input to the next step.

#### 2.2.2 Step 2 LDA modeling

LDA modeling is a two-part process (Blei et al., 2003; Rehurek and Sojka, 2010). In the first part of LDA, topics are generated by comparing word co-occurrence across the full corpus to identify latent topics present in the corpus and then create a topic model. This helps create an initial set of topics. In the second part of LDA, a document is compared to a previously created topic model, and the document is assigned a probability (score), representing the extent to which it is associated with each topic in the model. These probability scores help determine which topics are most relevant to a given document.

##### 2.2.2.1 Model generation

A critical aspect of LDA modeling is the careful selection of hyperparameters to balance thematic granularity, interpretability, and computational efficiency. In Binkley et al. (2014) underscore the necessity of informed and context-dependent parameter selection in LDA applications rather than universally applicable LDA parameter values. Following this principle of corpus-specific parameter selection, we empirically tested threshold values from 0.1 to 0.5 on both datasets. For the smaller patent corpus (350 documents), empirical testing revealed that higher thresholds were necessary to reduce noise and spurious topic assignments that were more prevalent in the smaller dataset. A threshold of 0.3 provided the optimal balance between meaningful topic assignments and document coverage. For the larger publication corpus (~130,000 documents), a threshold of 0.2 maintained broad coverage while preserving semantic relevance, as the larger sample size provided more robust topic-document associations.

We implemented LDA using the Gensim library (Rehurek and Sojka, 2010), a widely used framework for topic modeling in Python, and systematically tuned the following key parameters:

- Number of Topics: Determines the granularity of topics. We optimized this parameter iteratively, balancing meaningful groupings with interpretability.
- Alpha (document-topic distribution) and Eta (word-topic distribution): Control the sparsity of topic distributions. These parameters fine-tune how specific documents align with individual topics and how words are distributed across topics.
- Passes, Update Every, and Iterations: Impact model stability and convergence by controlling the number of revisions in topic assignments.
- Chunk size and Learning Method: Influence the efficiency and scalability of the model, particularly for large datasets.
- Random Seed: Set to ensure reproducibility.

To evaluate the effectiveness of our topic modeling approach and fine-tune the hyperparameters, we considered a combination of quantitative and qualitative evaluation metrics: We measured the semantic consistency of the topics using the coherence metric, u_mass, which evaluates the co-occurrence patterns of word pairs within topics. Supplementary Figure 5 shows the coherence score for different topic numbers in the publication data. While high coherence scores are often considered a sign of topic quality, we found that they do not always correlate with effective topics. In some cases, models with slightly lower coherence scores produced more distinct topics, which proved more useful for grouping related documents. The highest coherence scores for publication data using fixed hyperparameters were observed for topic numbers 250 and 300. Based on SME reviews of the topics, topic number 300 was determined to be the best fit for the publication data. Consequently, hyperparameters were initially selected based on coherence scores, and further fine-tuning was performed with input from SME. Reviews were conducted to ensure the topics were both semantically coherent and sufficiently distinctive for analytic clustering tasks, as well as the accurate representation of the underlying themes within the corpus.

We separately tuned the hyperparameters for each of the models that were created from the discrete document types: publications, projects, and patents. Supplementary Figure 6 shows an example of the final hyperparameter tuning for Project Dataset. The final hyperparameters used for each dataset are summarized in Table 1.

**Table 1:** Final hyperparameter configuration for the topic model from datasets publications, patents, and grants/projects.

**Model data source**	**Number of topics**	**Passes**	Update every^*^	**Iterations**	**Learning method**	**Chunk size**	**Alpha**	**Eta**	**Random state**
Publications	300	5	5	100	Online	10,000	0.5	0.0001	0
Patents	150	5	5	50	Online	20	0.1	0.0001	0
Grants/projects	100	1	1	50	Online	20	0.5	0.0001	0

* Update_every is the LDA hyperparameter that controls how often the model updates its parameters during training.

The output of LDA for each model includes a list of topics and the word-topic distribution matrix, which shows the probability of each word contributing to specific topics. This helps identify the most relevant terms for each topic and aids in the interpretation of the discovered topics. For interpretability, the top 10 most frequent words for each topic were extracted from the LDA model, providing key terms that define each topic. This is a widespread practice to make topics more interpretable (Blei et al., 2003). Additionally, we leveraged PyLDAVis outputs to identify the top relevant words for each topic (Sievert and Shirley, 2014), since the LDA model itself provides only the most frequent words.

##### 2.2.2.2 Application of models to datasets

Note that once a topic model is generated by the LDA algorithm from a corpus of documents, it can then be applied to the same corpus from which it was generated and the LDA model will assign a probability that each document discusses each specific topic in the model (Blei et al., 2003; Blei and Lafferty, 2009). A generated topic model, however, is not limited in application to solely the documents from which it was created. Any corpus of documents can be pre-processed and a model generated from a distinct set of documents may be applied to it. A document may be compared to any LDA model and be assigned a score for the topics the model contains.

The output of the application of an LDA model to a dataset is the document-topic distribution matrix. This provides the probability of each document being associated with each specific topic in the model. This allows us to understand how strongly a document relates to different topics, which is key for interpreting the thematic relevance of individual documents.

In the current work, we applied two topic models to the Grants/Projects dataset. We first applied the topic model generated from the Grants/Projects dataset itself to all the Grants/Projects documents. Next, we applied the LDA model, which was generated from the larger corpus of publications to the Grants/Projects dataset. We found that many of the documents contained latent topics that were explored in greater detail in the publications, which were more appropriate for analyzing benefits (see Section Results).

We also applied two models to the Publications data set, both the one generated on the Publications data (see Section Results below) and the one generated on the Grants/Projects data.

We applied only the Patents LDA Model to the patent data due to the prevalence of patent legal jargon in these documents and present the results below.

##### 2.2.2.3 Generation of short labels for each topic

Since listing large numbers of topic words in visualizations for subsequent data analysis can be counterproductive, having short meaningful labels for each topic facilitates labeling the topics in analytics and graphics. The top 30 most relevant words and top 30 most frequent words per topic were input into GPT-4.0, which generated a topic label. Guidelines for the prompts used in this process are given in Supplemental Table 1. An SME then validated and refined these labels before applying benefit tags. The ChatGPT prompt objective was to extract labels based on relevant and frequent words from the datasets with relevant words weighted higher. Each short label was then included in the spreadsheet alongside the relevant and frequent words for SME review described in **Step 3**.

#### 2.2.3 Step 3 tagging of topics and documents

##### 2.2.3.1 Human in the loop tagging of topics

Manual tagging of each topic with its appropriate benefits category by a SME is feasible since it occurs at the level of a few hundred or fewer topics, and not directly for each of thousands of documents. Using a spreadsheet that included the 30 most relevant words for the LDA topic, the 30 most frequent words for the LDA topic, the short labels generated by GPT-4.0, and the descriptions of TSBM benefits categories and subcategories from the latest revision of the TSBM (Luke et al., 2018), GPT-4.0 assigned initial TSBM tags and a SME reviewed each topic in the spreadsheet. A 1 (TRUE) tag was assigned to all relevant benefits categories in a designated column when the topic matched a benefit description. The SME also documented supporting terms from the relevant word list in another column and categorized the topic within the TSBM framework in a separate column to facilitate a secondary review of the assignments. A secondary review was conducted by spot-checking. This followed the same process as that described for the manual tagging but was performed by a second SME to ensure that the benefits assignments agreed.

##### 2.2.3.2 Manual review of short topic labels

During the Manual Tagging of Topics, the SME also validated the short labels and made corrections or refinements as needed.

##### 2.2.3.3 Automated tagging of documents

After completion of the manual reviewing and tagging, an automated process applied the TSBM benefit tags for a topic to all documents with a topic score of 0.2 or higher for that specific topic for Projects/Grants and Publications. A score of 0.3 was used for Patents accounting for the small number of patents present. By manually tagging just a few hundred topics and automatically transferring the tags to documents having those topics, a large corpus can be effectively tagged with expected TSBM model benefits assigned to each appropriate document.

#### 2.2.4 Step 4 analysis and visualizations of pipeline outputs

Analysis and visualizations are of primary importance in understanding the topic models, their application to the data and to evaluating the results of the automated tagging system. Using the word-topic distribution matrix output by the first part of LDA that shows the probability of each word contributing to specific topics helps identify the most relevant terms for each topic and aids in the interpretation of the discovered themes. We also leverage the topic distribution matrix output by the second part of LDA that gives the probabilities that each document has a topic. To explore and visualize these outputs, we utilized heatmaps, Principal Component Analysis (PCA) (Jolliffe, 2002), t-Distributed Stochastic Neighbor Embedding (t-SNE) (Van der Maaten and Hinton, 2008), and clustering techniques. Each of these methods provided complementary insights into the underlying structure of the data.

##### 2.2.4.1 Heatmap generation

We converted the topic distribution matrix from the LDA into a DataFrame to analyze the topic probabilities across individual documents. This DataFrame was further enriched by integrating metadata, such as PMID, year, and organization names. Including such metadata enables contextual analysis and allows us to observe how topics are distributed across distinct groups.

A heatmap was generated to visualize the distribution of topics across organizations or hubs. Min-max scaling was used to normalize the topic probabilities within the range of [−1, 1], making it easier to compare topics across documents. Hierarchical clustering (Ward's method, Euclidean distance) was applied to reorder columns in the heatmap to reveal patterns of similarity between documents or topics. This approach is well-suited for identifying hierarchical relationships between topics (Müllner, 2011).

##### 2.2.4.2 Visualizing topic clusters

Clustering was performed to visualize the LDA output. Although LDA is a soft clustering method, it can be difficult to interpret its results in high-dimensional data like publications and project corpora without further analysis. When applying the model to a dataset, the topic probability distribution table will give a score for every topic to every document, and therefore selecting a cutoff score for considering that the topic is significantly present in the document is a key, tunable feature.

We explored multiple methods for clustering documents based on their topics including PCA on LDA Topic Distribution, PCA on TF-IDF, K-means clustering, Naïve LDA (Sun, 2014), and LDA-Max. We selected LDA-Max, further supplemented with T-SNE, based on empirical exploration and spot-checking of clusters to ensure meaningful and cohesive groupings of documents, which was evaluated by comparing them to document titles and abstracts.

Under results, we show these LDA topic models applied to different datasets. The top 5 topics in projects/grants, patents, and publications were identified using the LDA-MAX approach, where each document was assigned to its most probable topic based on the highest topic probability score after the cutoff. This process is not needed to count the benefits but is useful for visualizing how the overall corpus is organized and what topics are part of its primary focus.

Notably, some topics encompass multiple subtopics and there may be nearly duplicated topics produced by the LDA. This requires tuning to balance increasing the number of topics to reduce compound topics while minimizing the redundancy of too many very closely related topics. We tested topic models with 25, 50, 75, 100, 150, 200, 250, 300, and 350 topics on publications. The 300-topic model provided a good balance, yielding distinct topics with minimal repetition yet does have subtopics in some of the topics.

##### 2.2.4.3 Inclusion of MeSH terms

The National Library of Medicine has developed an extensive system for assigning Medical Subject Headings (MeSH) terms to publications. This system is built upon a vast corpus of scientific literature, leveraging human curation and algorithmic methods to standardize topic categorization. MeSH terms serve as controlled vocabulary descriptors that categorize biomedical and health-related research, making them a valuable tool for contextualizing the impact of translational science. To enhance topic modeling outputs and explore whether additional insights could be gained, particularly in domain areas with fewer TSBM tags, we incorporated MeSH terms into select clustering visualizations when using publications as the dataset.

This supplementation was directed primarily to enhance subsequent analysis and exploration of the research outputs and specifically aimed to:

- Refine topic clusters by incorporating additional structured metadata
- Identify underrepresented areas in the TSBM framework by detecting Research Topics heavily represented in MeSH but with sparse TSBM tagging

MeSH terms were integrated into the clustering process in the following ways:

- Co-occurrence Analysis: We cross-referenced MeSH terms assigned to publications in our dataset with the topics generated by LDA. This quantified the overlap between NLP-identified themes and MeSH-annotated research categories.
- Topic Enrichment: After running LDA topic modeling, we mapped MeSH terms to the top words in each generated topic. The presence of MeSH terms in the generated topics validated topic coherence and identified areas where TSBM benefit tags were sparse but significant biomedical themes emerged.
- Identifying Additional TSBM Benefits: After running the LDA topic model, we mapped certain MeSH terms to the TSBM Benefits. The presence of these MeSH terms was used for comparison to assign TSBM Benefit tags. Comparisons between TSBM-tagged and MeSH-tagged research outputs could reveal areas where one method or the other identifies unique benefits and serve to reveal the overlap between the approaches.

### 2.3 Data sources and pipeline data flows

**Figure 2:**
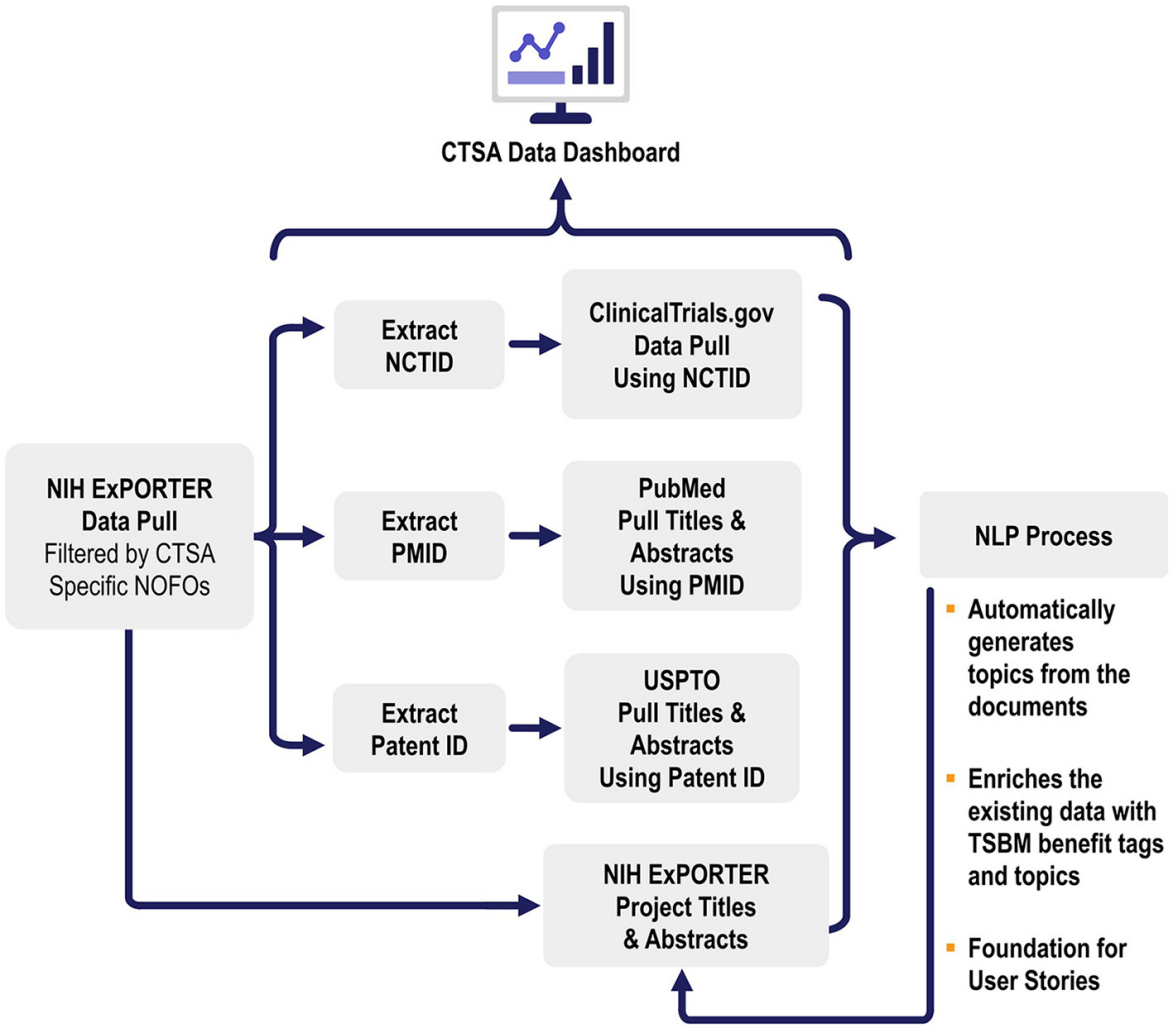
Data sources and data workflows. This figure illustrates the data workflows for automated translational science benefits model impact extraction. The process begins with data pulled from NIH ExPORTER filtered by CTSA-specific NOFOs, followed by the extraction of NCTIDs, PMIDs, and Patent IDs. These identifiers are used to pull data from ClinicalTrials.gov, PubMed, and USPTO, respectively. The extracted titles and abstracts are then processed using NLP and the results are used to enrich the pulled data.

This study utilizes three key datasets: Projects/Grants, publications, and patents, all integral components of the CTSA program (Figure 2). These datasets were selected for their relevance in identifying and tagging societal benefits in research, aligned with the CTSA program's mission to accelerate the translation of research into clinical practice. The titles and abstracts from each dataset were chosen due to their ability to succinctly summarize the core themes, goals, and findings of the research while enabling computational feasibility compared to the processing of entire documents.

#### 2.3.1 Publication data set

The data for publications were sourced using ExPORTER [National Institutes of Health (NIH), 2023], a publicly available resource that provides bulk administrative data from NIH RePORTER. CTSA-specific hub projects (National Center for Advancing Translational Sciences, 2024) were identified using Notice of Funding Opportunity Numbers (NOFOs) as filters, and publication IDs (PMIDs) linked to these projects were extracted through ExPORTER's link tables. Using these PMIDs, we obtained CTSA-specific publications, enabling the extraction of associated publication records. Since ExPORTER lacked the detailed textual data required for the topic analysis, we enriched the dataset using the PubMed API. This process added abstracts, MeSH terms, keywords, and other essential metadata, ensuring a more comprehensive resource. Quality control measures included removing duplicates and filtering publications based on organization names and activity codes.

Initially, publications were identified based on Notice of Funding Opportunity (NOFO) filters. After extracting metadata for publications and filtering by hub, activity code, and project-aligned publication years, we deduplicated records with identical titles and abstracts. The final dataset only included the deduplicated results.

#### 2.3.2 Patent data set

Patent data was sourced from ExPORTER by linking the NOFO-filtered project IDs to related patents. Metadata, including abstracts, titles, and filing dates, were retrieved through the PatentsView API. In alignment with the TSBM model, patents are considered a direct economic impact, as they represent tangible innovations that have the potential to lead to commercialization, contributing directly to societal and economic benefits.

Initially, patents were identified through project links. After filtering based on CTSA hub organization names and activity codes, and deduplicating records, only the patents that remained were included in the final dataset.

#### 2.3.3 Project/grants data set

The Project Data Set consisted of grant data that was sourced from ExPORTER and filtered by NOFOs. Key fields such as abstracts, titles, health relevance, and associated terms were included in the data set.

Grant abstracts were first identified, then filtered by hub-specific activity codes and organization names, and finally deduplicated to yield the final set of projects.

#### 2.3.4 Information from ClinicalTrials.gov

Although not directly included in the benefits extraction pipeline, CTSA-funded clinical trials information was extracted as meta-data.

## 3 Results

This section presents the outcomes of our NLP-driven benefit extraction pipeline, demonstrating how LDA topic modeling and automated tagging facilitate the identification of TSBM benefits across CTSA-funded research outputs spanning CTSA hub grants awarded from 2006 to 2023. The corpus comprised 127,958 publications and 352 patents linked to 1,296 grants. We first conducted a BoW analysis, which provides an initial thematic overview of CTSA research by highlighting frequent terms in Projects/Grants, patents, and publications. We then examine the application of LDA topic models on the datasets. We summarize the distribution of extracted benefits, detailing which categories, clinical, community, economic, and policy, were most prevalent and where the gaps remain. We compare the results of this topic-based analysis to the use of benefits identified with MeSH-based classifications to assess the strengths and limitations of each method. Next, we present topic trends using heatmaps to visualize how Research Topics align across the CTSA program and its hubs. Finally, we present the LDA topic modeling results, illustrating how different datasets group into distinct research themes and how these topics map to TSBM benefit categories.

### 3.1 BoW results

**Figure 3:**
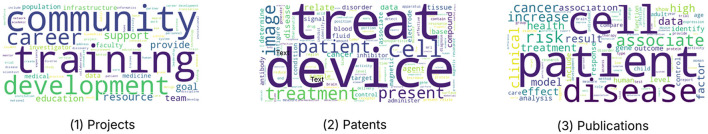
BoW Results. The visualizations presented in this figure illustrate the thematic focus of CTSA research across three distinct datasets: Projects/Grants, Patents, and Publications. Each word cloud highlights the most frequently occurring terms within the respective dataset, providing a visual summary of the key research themes and priorities.

The BoW analysis provides an initial thematic overview of the CTSA-funded datasets by identifying the most frequently occurring terms in different datasets. Figure 3 illustrates word clouds generated for projects/grants, patents, and publications. We find that the word frequencies accurately reflect the different purposes of these types of documents.

In the Projects/Grants dataset, the word cloud highlights key terms such as “community,” “training,” “development,” “career,” and “support,” reflecting a strong emphasis on workforce development, mentorship, and community engagement within translational science initiatives that reflects the purpose of the grants funded by CTSA. The Patents dataset is characterized by terms like “treat,” “device,” “cell,” and “treatment,” underscoring a focus on medical innovations, therapeutic interventions, and biotechnology advancements appropriate to a corpus of documents referencing novel inventions created with CTSA funding. The Publications dataset prominently features words such as “cell,” “patient,” “disease,” “cancer,” “association,” and “risk,” indicating a strong concentration on disease research, clinical applications, and patient outcomes as would be expected for scientific publications of research results in translational science. Collectively, these results illustrate the distinct yet complementary research priorities across CTSA-funded projects, patents, and publications.

### 3.2 LDA results

LDA topic modeling was applied to CTSA-funded projects, publications, and patents to identify thematic structures and research focus areas represented as topics. Three distinct LDA models were generated, each optimized for its respective input dataset and tested for applicability on both its own and other relevant datasets. Here we present in detail the resulting LDA models including their top topics and topic distributions.

We generated three LDA topic models from these datasets:

- Projects/Grants LDA Model, generated from the titles and abstracts of NCATS funded grant applications aligned well with NCATS and CTSA strategic goals but proved less useful for TSBM benefit identification due to their high-level nature. As might be expected for a grant application, many of these abstracts focused on the core mission of the CTSA and the topics identified in these documents reflect that.
- Publications LDA Model, generated from titles and abstracts of CTSA-linked publications, produced detailed, domain-specific topics, many of which aligned directly with TSBM categories and subcategories. The greater scientific detail present in journal abstracts combined with far larger numbers of publications than project abstracts, enabled the Publications model to have a rich set of research-related topics.
- Patents LDA Model, generated from title and abstracts of CTSA-linked patents, captured the distinct vocabulary and purpose of patents compared to scientific publications. Unsurprisingly, given the highly specific nature of patents and their legal requirements, this model was more focused on specific invention description language, pragmatic utility than the model derived from publications

**Figure 4:**
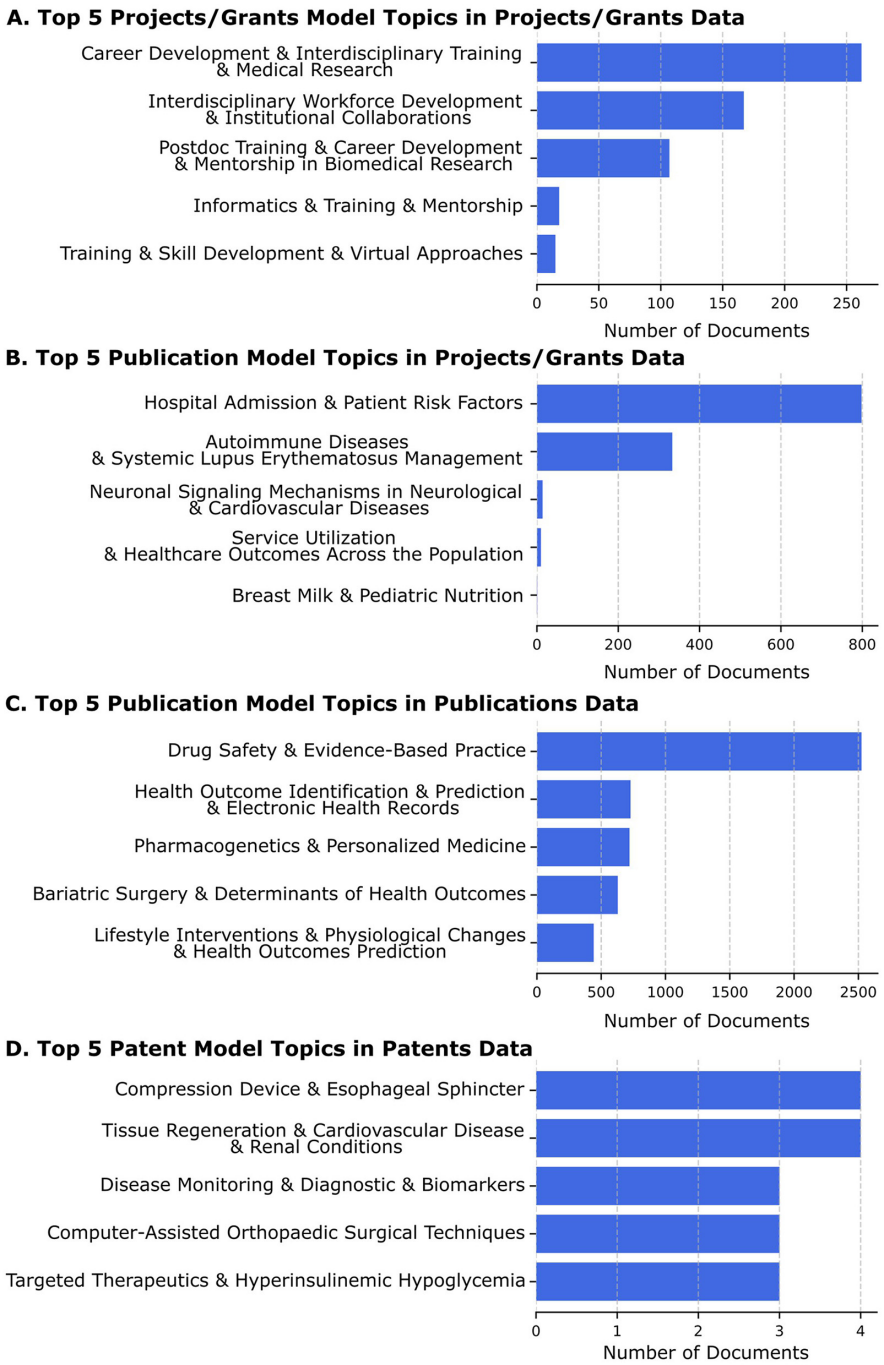
The top 5 topics for each model application to a dataset are shown, along with a bar graph of the number of documents assigned to that topic with a high probability by the LDA model on that corpus. The labels shown are the short ones derived from GPT4.0. **(A)** The top 5 patent model topics found in projects/grants are based on the highest LDA probability scores. The leading topic, “Career Development and Interdisciplinary Training and Medical Research” has over 250 grant abstracts assigned. **(B)** The top 5 publication model topics seen in projects/grants are based on the highest LDA probability scores. The leading topic, “Hospital Admission and Patient Risk Factors” has over 800 projects associated with it based on LDA probability scores. It is an example of a compound topic that still includes two related topics, both of which would be expected to be well-represented in translational science grant applications. **(C)** The top 5 publication model topics assigned to publications based on the highest LDA probability scores. The leading topic, “Drug Safety and Evidence-Based Practice” has about 2,500 publications associated (out of the more than 100,000 in the corpus) with it based on LDA probability scores. It appears to be an appropriate topic that would be expected to be found in many translational science research publications. **(D)** The top 5 patent model topics assigned to patents based on the highest LDA probability scores. The leading topic is found in just 4 of the 352 patents, not surprising given that a patent by its nature is expected to describe a unique invention.

In Figure 4, we show these LDA topic models applied to different datasets

We applied the Projects/Grant Model to Projects/Grant Data (see Figure 4A). We found that this revealed the projects (grant abstracts) aligned well with NCATS and CTSA strategic goals but were not as useful for TSBM benefits (see Figure 4A). The leading topic, “Career Development & Interdisciplinary Training & Medical Research” has over 250 project/grant documents assigned to it based on the probability scores, highlighting its prominence, it also appears to be a compound topic, with each subtopic likely to be well-represented in a translational science grant application. We also applied the Projects/Grant Model to the Publications data but also found this did not facilitate the identification of TSBM benefits (data not shown).

To achieve improved alignment with TSBM, we examined the Projects/Grants data set using the model we generated using the Publications data (see Figure 4B). This process was able to identify many TSBM benefit related topics. The leading topic, “Hospital Admission & Patient Risk Factors” has just under 800 grant abstracts associated with it based on LDA probability scores. This topic seems appropriate for an analysis of applications to conduct clinical research in accordance with the NIH Opportunity descriptions, and it would be expected that most Project/Grant titles and abstracts would include this topic.

We next applied the Publications Model to the publications data itself, and this produced a wealth of highly relevant topics, showing the publications data to be a rich source of potential TSBM benefits (see Figure 4C). The leading topic, “Drug Safety & Evidence-Based Practice” has about 2,500 publications (out of the 100,000+ publications) associated with it based on LDA probability scores. This topic is also expected to be well-represented in a corpus of publications resulting from translational science research.

Finally, we applied the Patents Model to the patents data. Since the language used in patent applications is very specific for patent legal requirements and uses common jargon, this model was the only one that we tested on the patent data (see Figure 4D). The leading topic, “Compression Device & Esophageal Sphincter” has 4 associated patents. The entire corpus of patents is 352 documents, and a patent is expected to be unique, so a much lower number of patents sharing similar topics is also expected by the documents. The method can still serve to identify small groups with related topics that may highlight advancements in translational science within a highly focused medical area.

### 3.3 Automated extraction of the TSBM benefits and summary counts

Following the generation of the topics, their association with benefits tags, and the assignment of tags to each document based on its topics, counts were made of each of the types of benefits identified. The results indicate that clinical and community benefits were most effectively identified using the Publication Model applied to Projects/Grants and Publications data. We note that while grant applications discuss benefits, these are potential benefits since the project/grant abstract is proposing a research approach rather than reporting on research accomplishments, while publication data reflects research that has been conducted with benefits partially or completely realized.

While economic benefits were less prominent in Projects/Grants and Publications data, all 352 Patents were tagged with Economic Benefit, as Patents are inherently classified as such under the TSBM framework. In addition, clinical benefits were identified in 87 Patents shown in Table 2.

**Table 2:** Summary of total number of documents tagged with TSBM potential benefits.

**Benefit category**	Projects/grants^*^	**Patents**	**Publications**
Clinical	1,158	87	6,380
Community	546	0	5,874
Economic	14	352^**^	638
Policy	517	2	419

* Refers to the Publication Model applied to Projects/Grants data. Note that the tagged documents do not represent direct TSBM benefits, as they originate from grant applications via NIH ExPORTER and instead reflect research aspirations rather than realized outcomes.

### 3.4 MeSH term comparison

**Figure 5:**
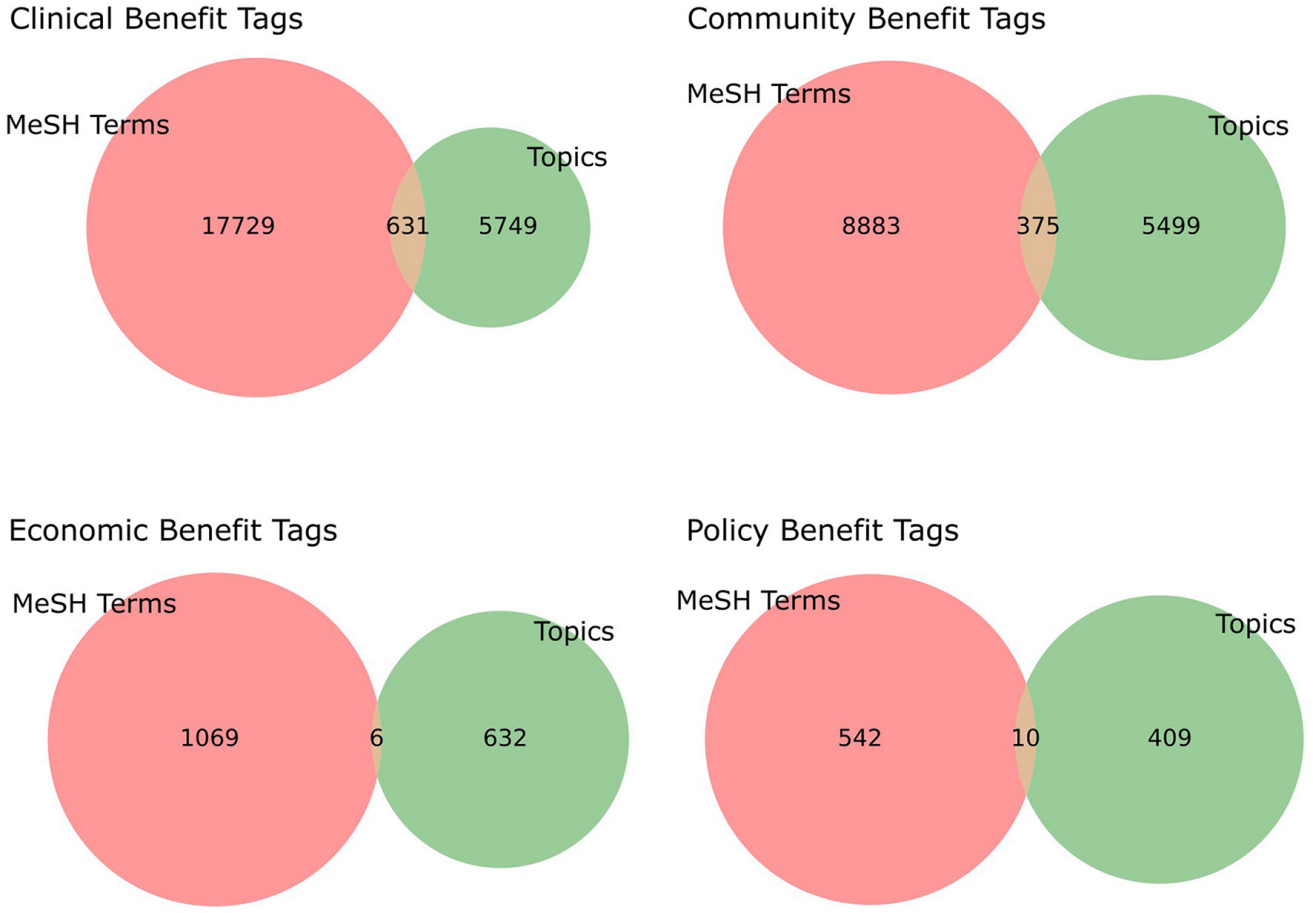
A Venn diagram of the publications found to contain TSBM-related MeSH terms compared to the automated TSBM benefits tags on Publication data. The majority of the publications found to have TSBM specific benefits identified with the automated process are not identified using MeSH terms. Further investigation is required to see if the publications containing a TSBM-related MeSH term in fact describe a benefit that fits the TSBM.

We selected a subset of MeSH terms that were related to TSBM to examine how their use compares to the NLP pipeline. Figure 5 shows that many of the benefits captured by the LDA-extracted topics are not captured by the MeSH terms. This may be due to the structured nature of MeSH classifications, which are based on a broad range of scientific publications, whereas NLP-driven insights are more directly tied to the actual outputs of the CTSA program. Although many publications may contain MeSH terms that could suggest potential benefits, their relevance has not been systematically verified. Specifically, it remains unclear whether the presence of a single MeSH term provides sufficient evidence that a publication contains a benefit that aligns with the TSBM. Verifying this is beyond the scope of the current project.

### 3.5 Overview of topic to CTSA hub organization relationships

**Figure 6:**
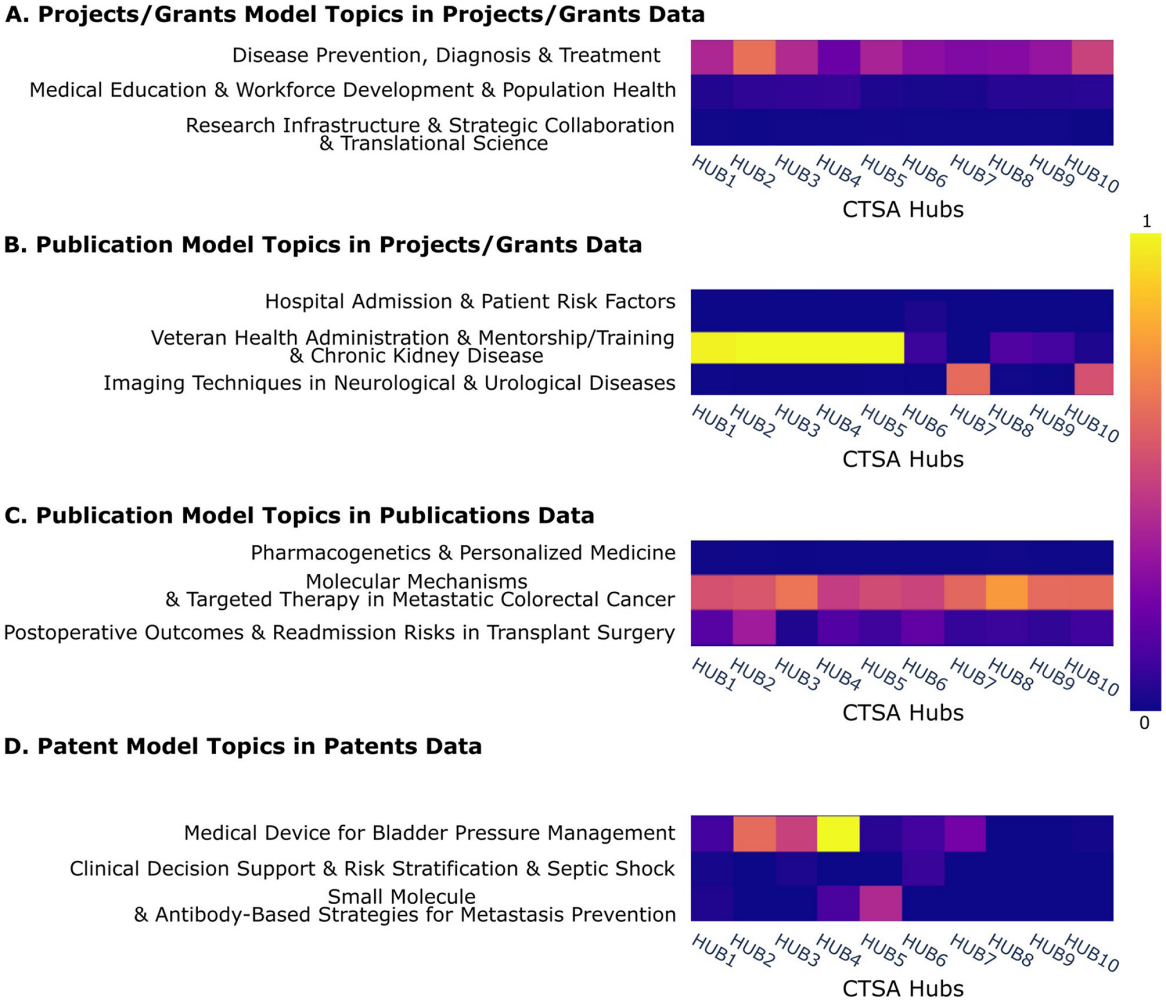
Heatmaps of LDA topic distributions across CTSA Hubs. **(A)** Project/Grants model topics in Projects/Grants data, **(B)** Publication model topics in Projects/Grants data, **(C)** Publication model topics in Publications data, **(D)** Patent model topics in Patents data, showing topic frequency for three example topics across 10 example organizations named anonymously. Blue indicates low topic prevalence in a hub, while yellow represents high prevalence. Full figures displaying the complete dataset can be found in Supplementary Figures 1–4.

The heatmaps in Figure 6 visualize the distribution of topics across CTSA hubs, providing insights into research focus areas and the concentration of specific themes within organizations. The y-axis shows three selected examples of topics, while the x-axis corresponds to 10 example hub organizations (anonymized), demonstrating the possibility for a comparative analysis of the prevalence of topics across institutions. A full set of topics vs. CTSA hubs is shown in Supplementary Figures 1–4.

Bright rows indicate ubiquitous topics that are widely represented across multiple hubs, while bright segments within an otherwise rather dim row highlight institution-specific focus areas. Conversely, dark rows signify topics with less emphasis across all hubs, reflecting areas of limited engagement. These patterns offer a data-driven perspective on institutional research strengths and thematic priorities within the CTSA program. Shared topics likely indicate potential or existing research collaborations.

Compared to the projects and publications topics that include many widely shared among hubs, the patents show more unique focus areas as would be expected for the development of a unique, patentable invention.

### 3.6 t-SNE projections of LDA topics across CTSA datasets

An alternative to looking at heatmaps to identify shared topics, is to look at how each data item (project, publication, or patent) clusters with other data items based on topic similarities. t-SNE projections were used to visualize thematic relationships within each dataset and across LDA models. Each point in the figures represents a document, colored by its most prominent topic, with large clusters therefore visualizing the most predominant topics in each corpus.

#### 3.6.1 Project/grant dataset analyzed with the projects/grants LDA topics model

**Figure 7:**
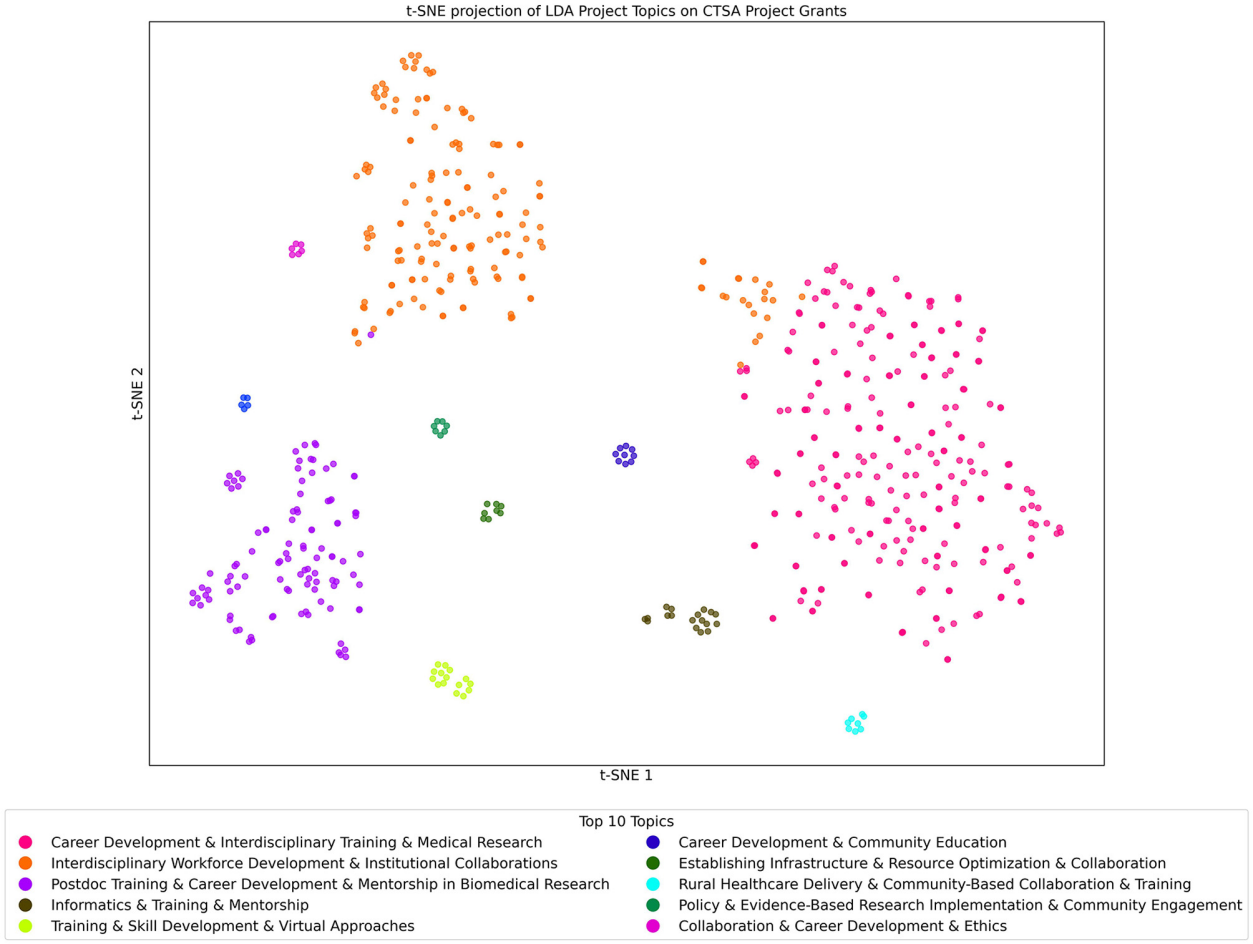
t-SNE Projection of LDA Topics of Projects/Grants Dataset Analyzed with the Projects/Grants Topic Model. Each point in the figure represents a project grant that is color-coded by its most prominent topic. Clusters reveal closely related projects, and the key gives the list of the top 10 topics found within the Project/Grants model when applied to the Projects/Grants dataset.

The t-SNE projection of LDA Projects/Grants Topics on CTSA Projects/Grants data shown in Figure 7 visually captures the program's thematic landscape. Using LDA with 100 topics, we identified key research areas, with each point in the t-SNE plot representing a project grant, color-coded by its most prominent topic. The clustering reveals groupings of similar Projects based on their most dominant topic, highlighting areas of concentrated research efforts. Notably, grants related to “Informatics & Training & Mentorship” (brown) and “Career Development & Community Education” (blue) form distinct, dense clusters, suggesting strong thematic coherence indicative of close similarity among these grants with respect to this topic. In contrast, topics such as “Interdisciplinary Workforce Development and Institutional” (orange) appear more dispersed, potentially reflecting that while a large group of grants discuss this topic, they do so with different emphasis. These patterns illustrate the diverse yet interconnected nature of CTSA-funded research.

#### 3.6.2 Project/grant dataset analyzed with the publication LDA topics model

**Figure 8:**
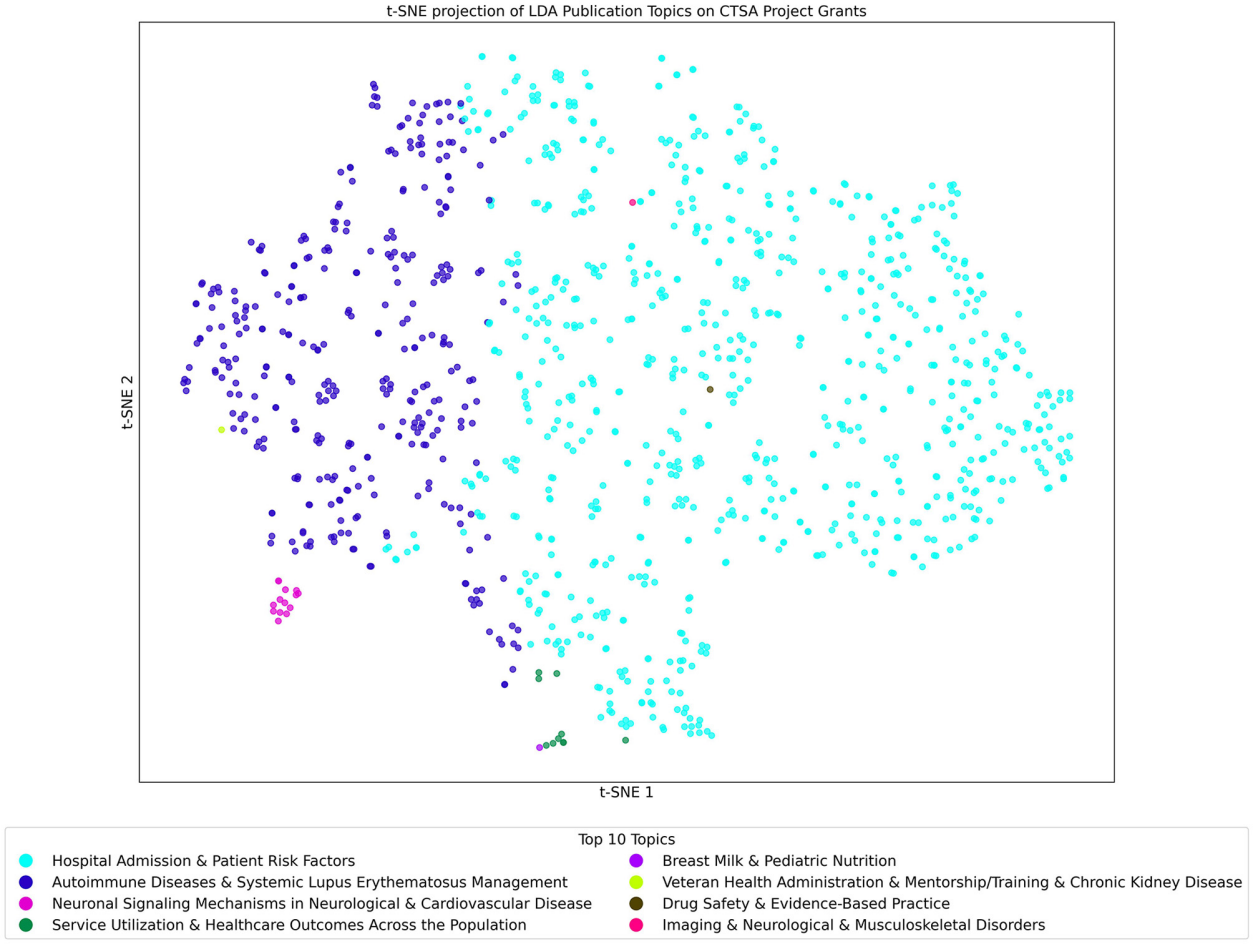
t-SNE Projection of LDA Topics of Project/Grant Dataset Analyzed with the Publication Topic Model. Each point in the figure represents a project grant that is color-coded by its most prominent topic. Clusters reveal closely related projects, and the key gives the list of the top 10 topics found within the Publication model when applied to the Projects/Grants dataset.

The t-SNE projection of LDA Publication Topics on CTSA Project/Grants shown in Figure 8 offers a detailed view of the program's research outputs, highlighting key areas of focus in contrast to the broader project topics. Using LDA with 300 topics, this visualization maps each project as a point, color-coded by its dominant Research Topic.

The clustering reveals distinct topic groupings, showcasing concentrated research efforts. The two most dominant topics across multiple projects are “Hospital Admission & Patient Risk Factors” and “Autoimmune Diseases & Systemic Lupus Erythematosus Management.” These topics, initially identified in publications, also appear prominently in the project data, indicating their widespread relevance and significance in CTSA-funded research. Each grant likely references additional specialized topics but, in this figure, we are only highlighting the single most predominant topic for each grant.

This analysis highlights that while these topics are present in the grant data, their significance becomes evident only through the publication-based model. They did not reach a level of significance in the Grants/Projects model. The use of the publication model offers a more nuanced perspective on CTSA-funded research, revealing both well-established areas and emerging fields that may not be as prominent in project grants. The model uncovers the interconnections between themes, demonstrating the program's role in supporting both ongoing research and innovative, specialized studies.

#### 3.6.3 Publication dataset analyzed with the publication LDA topics model

**Figure 9:**
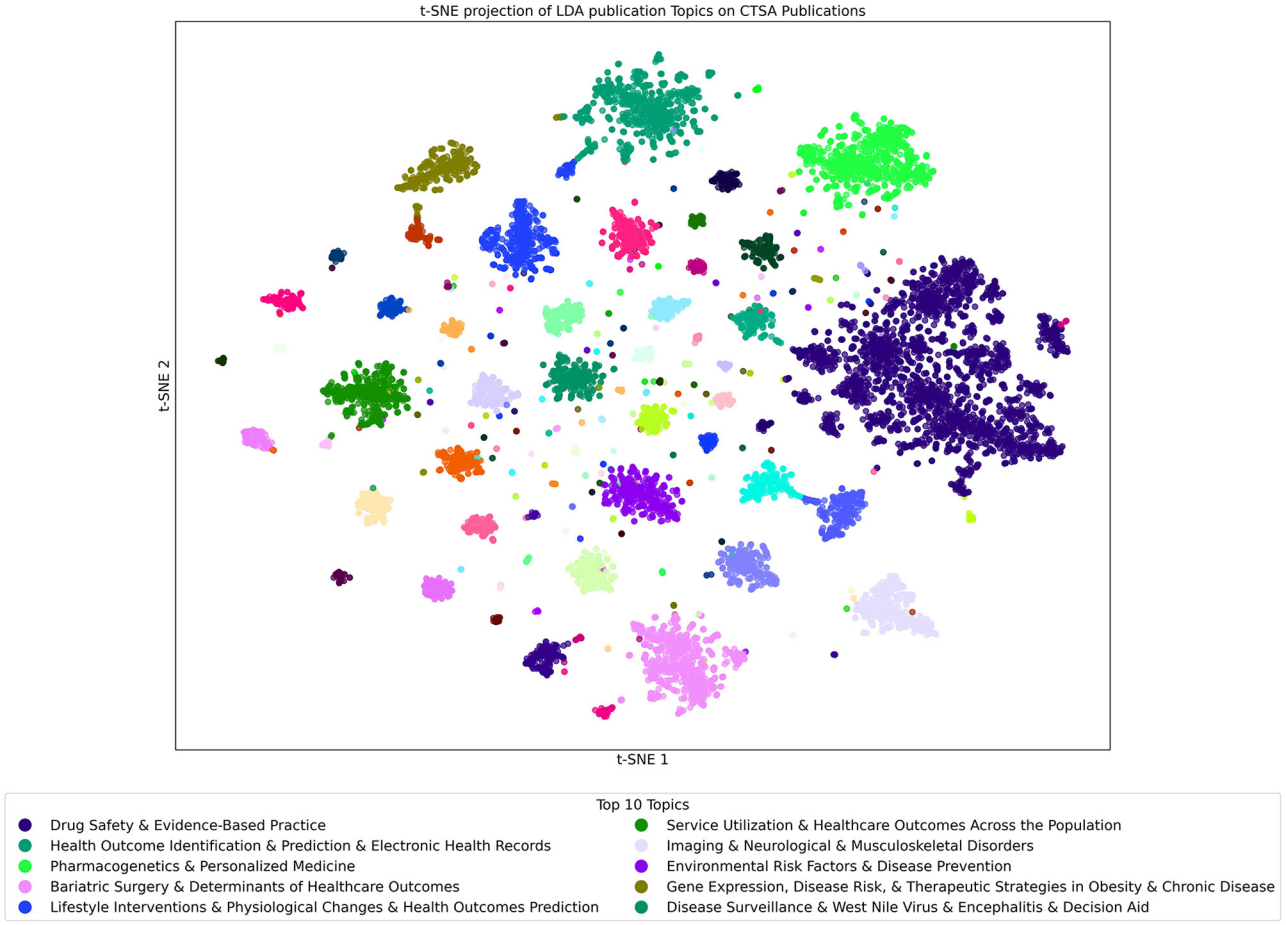
t-SNE Projection of LDA Topics Built on Publication Dataset Analyzed with the Publication LDA topic model. Each point in the figure represents a publication that is color-coded by its most prominent topic. Clusters reveal closely related publications, and the key gives the list of the top 10 topics found within the Publication model when applied to the Publications dataset.

The t-SNE projection of LDA Publication Topics on CTSA Publications as shown in Figure 9 offers a snapshot of the program's vast research output, covering over 130,000 publications. Using LDA with 300 topics, this visualization provides a detailed mapping of research themes, with each point representing a publication color-coded by its dominant topic.

Due to the large number of publications covering a wide range of topics, the data forms many clusters of closely associated publications. Several topics are dominant, with “Drug Safety & Evidence-Based Practice” being the most widely observed, clearly aligning with the goals of translational science. Other highly prevalent topics include “Health Outcome Identification & Prediction & Electronic Health Records” and “Pharmacogenetics & Personalized Medicine,” which emphasize advancements in predictive healthcare and personalized treatments. Beyond these larger clusters, even the smaller ones reveal additional specialized topics, further demonstrating the breadth of research in the CTSA Program. While some relevant topics are not shown here due to highlighting only the most dominant topic in each publication and only the top 10 topics for the model, the additional topics still contribute to the overall impact of the research landscape.

#### 3.6.4 Patent dataset analyzed with the patent LDA topics model

**Figure 10:**
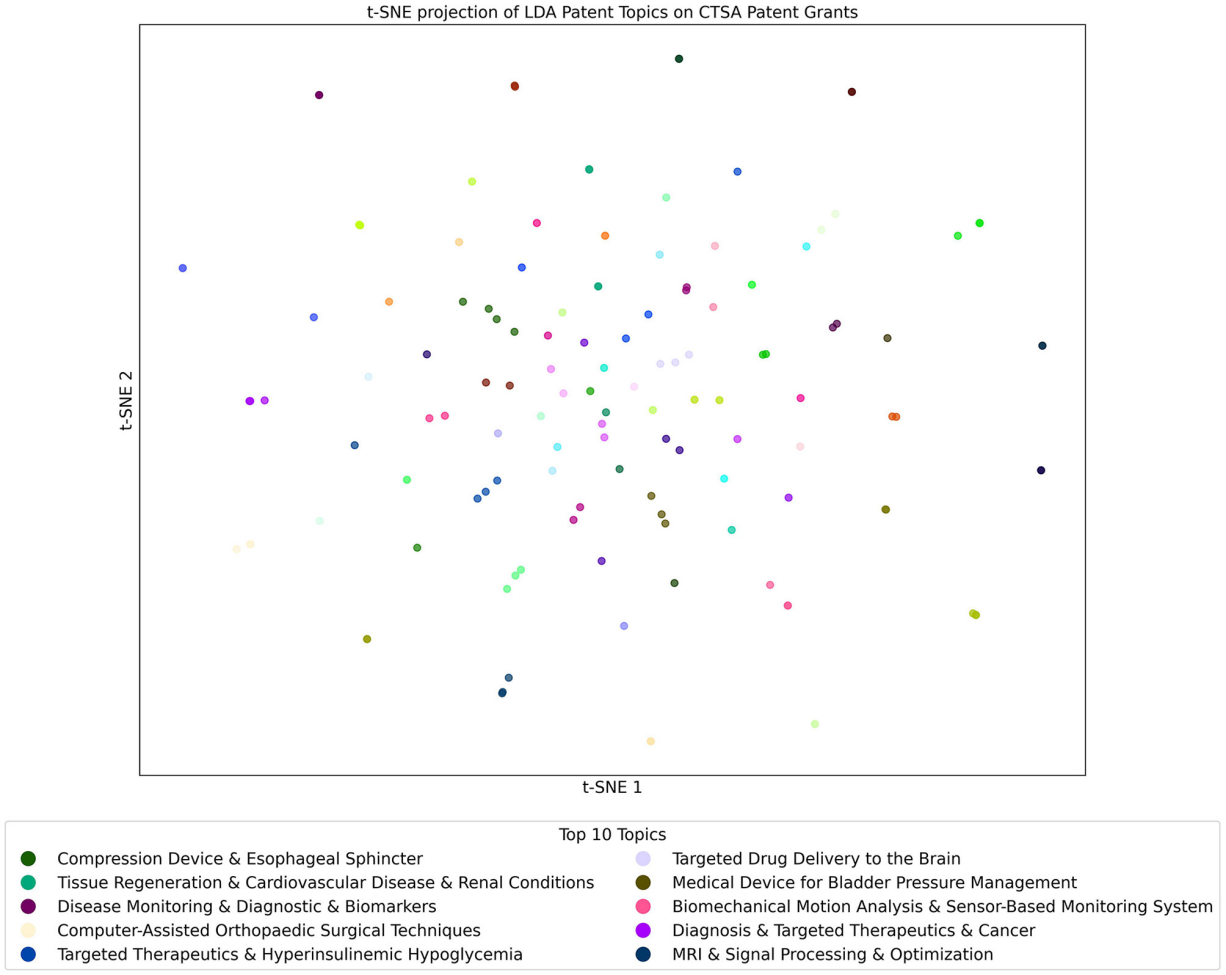
t-SNE Projection of LDA Topics from the Patents Topic Model used on the Patents Dataset. Each point in the figure represents a Patent that is color-coded by its most prominent topic. Clusters reveal closely related patents. The key shows the top 10 topics from the model that are found in the Patent documents.

The t-SNE projection of LDA Patent Topics on CTSA Patents as seen in Figure 10 shows a focused landscape of technological innovation and medical advancements. The figure shows a distribution of patents where no single topic stands out as dominant, which is consistent with the observation in Figure 4, where the leading topic appeared in only four documents. This aligns with the inherently specialized nature of patents indicating a diverse range of innovative efforts across multiple domains, reflecting the program's broad support for pioneering technologies in healthcare. The topics shown in the figure tend to be more unique and specific, further underscoring the focused and specialized nature of patent-related research within CTSA-funded projects. In summary, this pattern reflects the targeted and niche nature of the topics present in patents while confirming that, as expected for unique inventions, only a few examples of shared topics exist.

## 4 Discussion

By automating the application of the TSBM to CTSA-funded research outputs, this NLP-driven approach enhances both the efficiency and scalability of translational research impact analysis. Swanson et al. reported that completing a single case study required ~9 h, which aligns with our own manual case study experience. Extrapolating this effort across the full corpus of CTSA-funded outputs would require thousands of hours of SME time (Swanson et al., 2025). By applying LDA topic modeling to publications, patents, and project grants, we identified their innate topics and mapped them to the TSBM framework reducing SME time to reviewing the hundreds of topics instead of the hundreds of thousands of documents. This approach augments bibliometrics, extracts a considerable amount of additional benefit information that is complementary and larger in scope and quantity to that found with MeSH terms, and enhances our understanding of how translational research contributes to public health, clinical advancements, policy changes, and economic benefits. To our knowledge, it is the first attempt to apply the TSBM at the scale of an entire program with over a hundred thousand research outputs.

The option to leverage LDA over trained methods such as SVM analysis was essential due to the lack of available training data for such machine learning tools. While a number of case studies using the TSBM have been published, their numbers are too small to support an accurately trained learning machine across all the variations of the TSBM benefits categories and the differing types of subcategories that can lead one of the four major benefits types. A methodology such as LDA that allows the topics to emerge from the corpus of documents, and the utilization of human expertise to match those topics to the TSBM benefits categories enabled a feasible approach to automation.

This bottom-up, data-driven approach enables the data itself to reveal trends and impacts, that can be applied to the CTSA program outputs as a whole and does not require directed queries or require preconceptions for guiding data mining. This greatly enhances the ability of the method to find new and emerging trends and benefits for the CTSA program overall.

Our findings indicate that clinical and community benefits were well-represented in publications and projects, reflecting the CTSA program's strong emphasis on patient-centered and community-driven research. In contrast, economic and policy benefits were extracted less frequently, leading to the inclusion of patents to supplement the information on economic benefits. In the future, we are exploring additional sources of policy-related documents to improve coverage of this area. The ability to systematically map LDA-derived topics to the TSBM framework provides a novel approach for measuring and visualizing the impact of translational science at scale.

Our approach integrates subject matter expertise into assigning benefit tags to the extracted topics while the initial topic modeling is automated. The expert can then focus on a manageable set of topics, which renders the review process feasible and efficient. The integration of subject matter expertise ensures that the assigned benefits accurately reflect the nuances of each topic, enhancing the reliability of the automated analysis. Subsequent tagging of large volumes of documents with the tags for each of its topics further enables automation of one of the most insurmountable steps for manual application.

This NLP approach is statistically reproducible and minimizes the risk of errors associated with more opaque methods, such as those involving LLMs prone to generating hallucinations (Vaswani et al., 2017) and ensures that the output accurately reflects the content of the documents themselves. We do introduce an LLM for generating short labels for topics, while the main extraction of the topics from the documents rests exclusively on the LDA.

While NLP-driven benefit extraction is well-suited for identifying program benefits and trends, caution is advisable for its use of comparative evaluation methodology. Since topics emerge by being present across multiple documents, more unique contributions and novel improvements in translational science methods can be overlooked or shown as less prominent topics and these gaps are disproportionately important in evaluating the outputs of individual grants. It also implies that the counts of TSBM benefits on the documents as a whole likely miss some potentially important but less common types of benefits.

### 4.1 Choice of models for each dataset

To achieve improved alignment with TSBM, we examined the Projects/Grants data set using the model we generated using the Publications data (see Figure 4B). This process was able to identify many TSBM benefit related topics, likely because the benefits topics were latent in the grant abstracts but had less emphasis in the text than the overall CTSA program goals and therefore did not surface as major topics in the model. However, when these latent topics were developed in detail in scientific publications, those topics could be found in the grant abstracts. We do note that while this is a useful exercise to identify what benefits are discussed in the grant abstracts, they are anticipated but not realized benefits since they occur at the time of funding application, not because of finalized research.

### 4.2 Leveraging MeSH terms

To complement the TSBM benefit extraction, we incorporated MeSH terms from PubMed. Results indicate that MeSH terms identified some benefits not captured by NLP, while our automated system extracted many benefits not identified by MeSH. This complementary nature is likely due to the MeSH system being trained on a broad scientific corpus that includes full-text publications, whereas our LDA-based system is tailored specifically to CTSA-funded documents using only titles and abstracts.

For maximizing benefit extraction, leveraging both approaches together can be important: incorporating MeSH terms for structured biomedical categorization while utilizing NLP-driven topic modeling to uncover translational science themes unique to CTSA-funded research.

### 4.3 LDA topic scores and considerations for benefit tagging

Current TSBM benefit tagging applies labels to topics and links them to documents. LDA assigns probability scores to indicate the strength of affiliation between topics and documents, and these scores vary based on dataset size. Careful interpretation of these scores is necessary to ensure that TSBM benefit tags are applied to the topics most likely to be present in a document. Using a relatively low LDA topic score can cast a wide net to capture all potential benefits but may raise the risk of false positive results, while the use of a more stringent score ensures that benefit tags are not given to documents that are only peripherally related to the topic that corresponds to a benefit and reduces the possibility of false positive results. For the study's purposes, we used a relatively stringent topic score, potentially erring on the side of missing some benefits due to false negative errors. Moreover, the inclusion of the human-in-the-loop for the assignment of benefits tags to topics, should largely eliminate these types of errors for that key step and its subsequent application of the each of the documents with a significant score for the topic.

For some uses, such as identifying groups of documents that might contribute information about a trend or research focus, it may be desirable to set a less stringent score in order to cast a wider net and not miss potentially related documents. Further analysis could then be done by subcluster analysis to generate user stories or compelling research narratives. This flexibility in the pipeline enables its use to both broadly identify benefits as well as to explore refined research groupings and it can distinguish broad thematic trends from specific, demonstrated program outcomes. The ability of NLP-driven tools to measure and communicate translational research outcomes and trends at scale is currently not well-addressed by any other methods.

### 4.4 Future directions

This study focused on directly linkable and publicly available data, such as grant applications, patents, and publications, which are explicitly connected to CTSA-ing. While this approach enables structured and systematic benefit extraction, it does not yet capture broader downstream impacts, such as policy adoption, clinical implementation, or long-term public health outcomes. We also acknowledge that there may be some omissions in the completeness of data identified primarily through NIH Reporter. However, the pipeline, despite these limitations, has enabled the first feasible examination of the TSBM applied to the CTSA program overall its lifespan and all of its hubs and represents a significant advancement over being strictly limited to the manual application of the TSBM model.

Future efforts expand beyond direct research outputs to incorporate downstream translational impacts by integrating additional data sources, including Policy citations from private commercial databases such as Overton to assess how CTSA-funded research informs policy and regulatory frameworks. Use of the pipeline for a new set of documents, such as those potentially obtained from the Overton database, is a straightforward application of the pipeline from data pre-processing through benefits tagging.

Clinical practice guidelines to evaluate how research findings translate into clinical decision-making and healthcare interventions. Further exploration of other datasets and methodologies will be required to enhance our understanding of how CTSA-funded research translates into real-world applications over time.

## 5 Conclusion

This study demonstrates the feasibility of NLP-driven automation in systematically identifying and classifying translational science benefits at scale for over 100,000 documents representing research outputs. Moreover, it allows the benefit topics to emerge from the corpus of documents without directed searching or preconceived notions of benefits that may be present. By applying LDA topic modeling, TSBM tagging, and complementary validation techniques, we successfully extracted thousands of translational benefits from CTSA-funded research outputs. By continuing to refine and expand NLP-based methodologies, we aim to enhance the ability to measure, analyze, and communicate the true impact of translational research, providing actionable insights for funders, researchers, and policymakers within the CTSA network.

## Data Availability

Publicly available datasets were analyzed in this study. This data can be found through the following links: https://reporter.nih.gov/exporter, https://pubmed.ncbi.nlm.nih.gov/download/, https://data.uspto.gov/home; https://clinicaltrials.gov/data-api/api.
