# Supplementary material for: Topic analysis on publications and patents toward fully automated translational science benefits model impact extraction
Source: Front Res Metr Anal. 2025 Sep 23;10:1596687. doi: 10.3389/frma.2025.1596687 (PMC12500706; doi:10.3389/frma.2025.1596687)
Supplement: Supplementary file 1 [file Data_Sheet_1.docx]

Supplementary Material

# Supplementary Figures and Tables

## Supplementary Figures

| 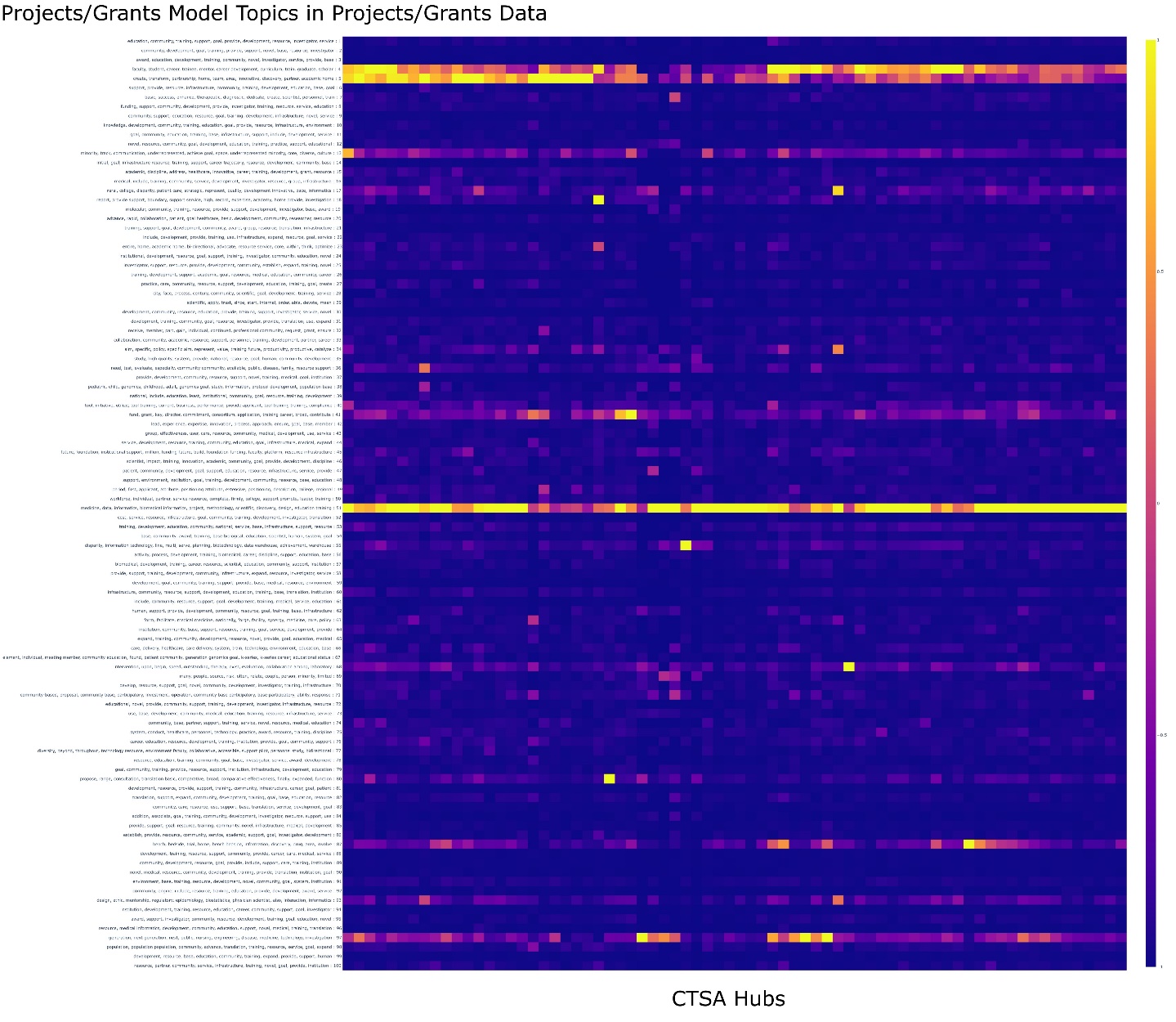 |
| --- |
| **Supplementary Figure 1:** Heatmap visualizing the distribution of Projects/Grants Model Topics on Projects/Grants Data across organizations/hubs. The topic distribution matrix from the LDA model was used to generate this heatmap. Blue indicates low topic prevalence in a hub, while yellow represents high frequency. Please refer to Supplementary_Fig1_Project_Topics_by_Hubs.jpg for a version with higher resolution. |

| 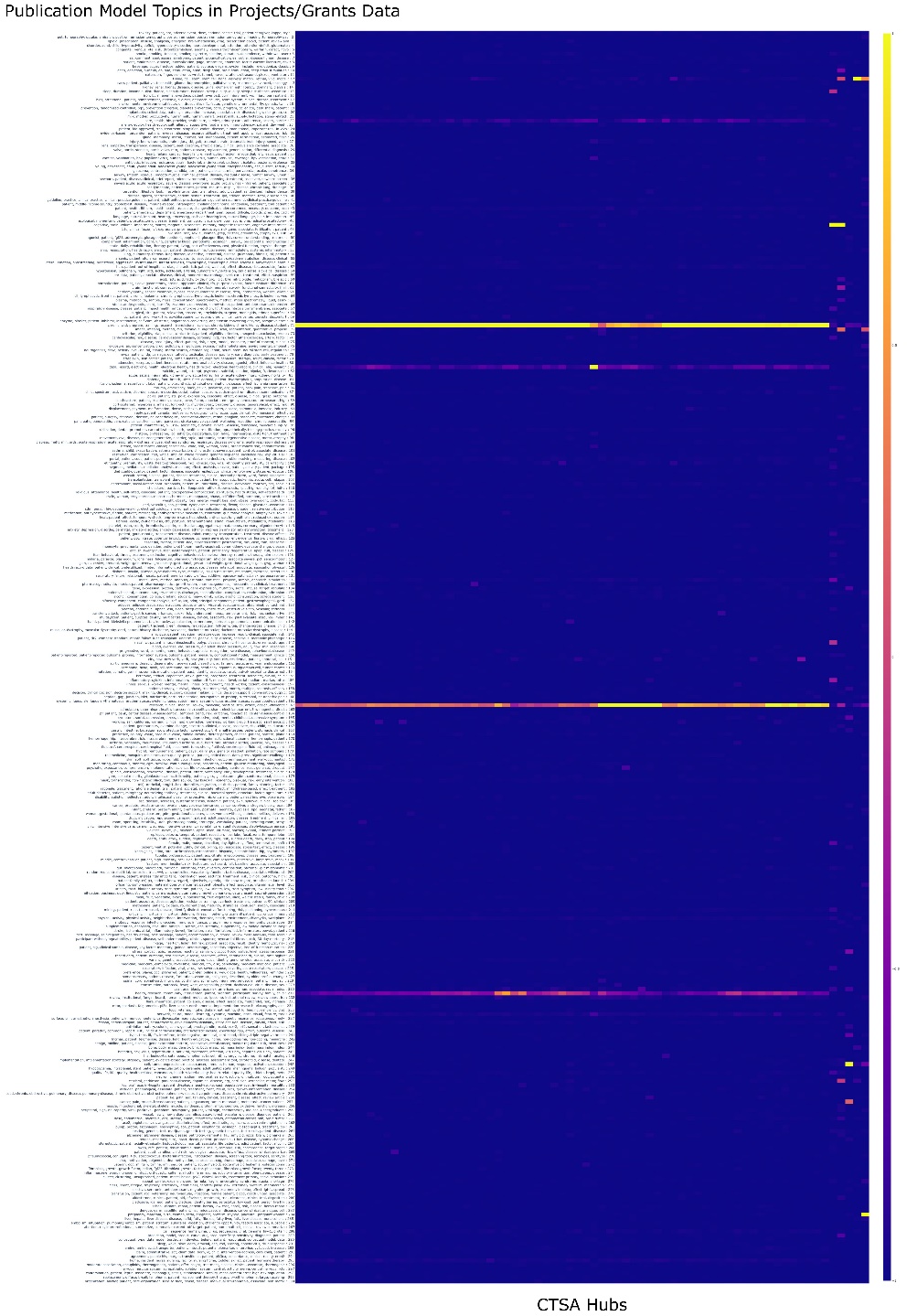 |
| --- |
| **Supplementary Figure 2:** Heatmap visualizing the distribution of Publication Model Topics on Projects/Grants Data across organizations/hubs. The topic distribution matrix from the LDA model was used to generate this heatmap. Blue indicates low topic prevalence in a hub, while yellow represents high frequency. Please refer to Supplementary_Fig2_Publication_Model_Project_Topics_by_Hub.jpg for a version with higher resolution. |

| 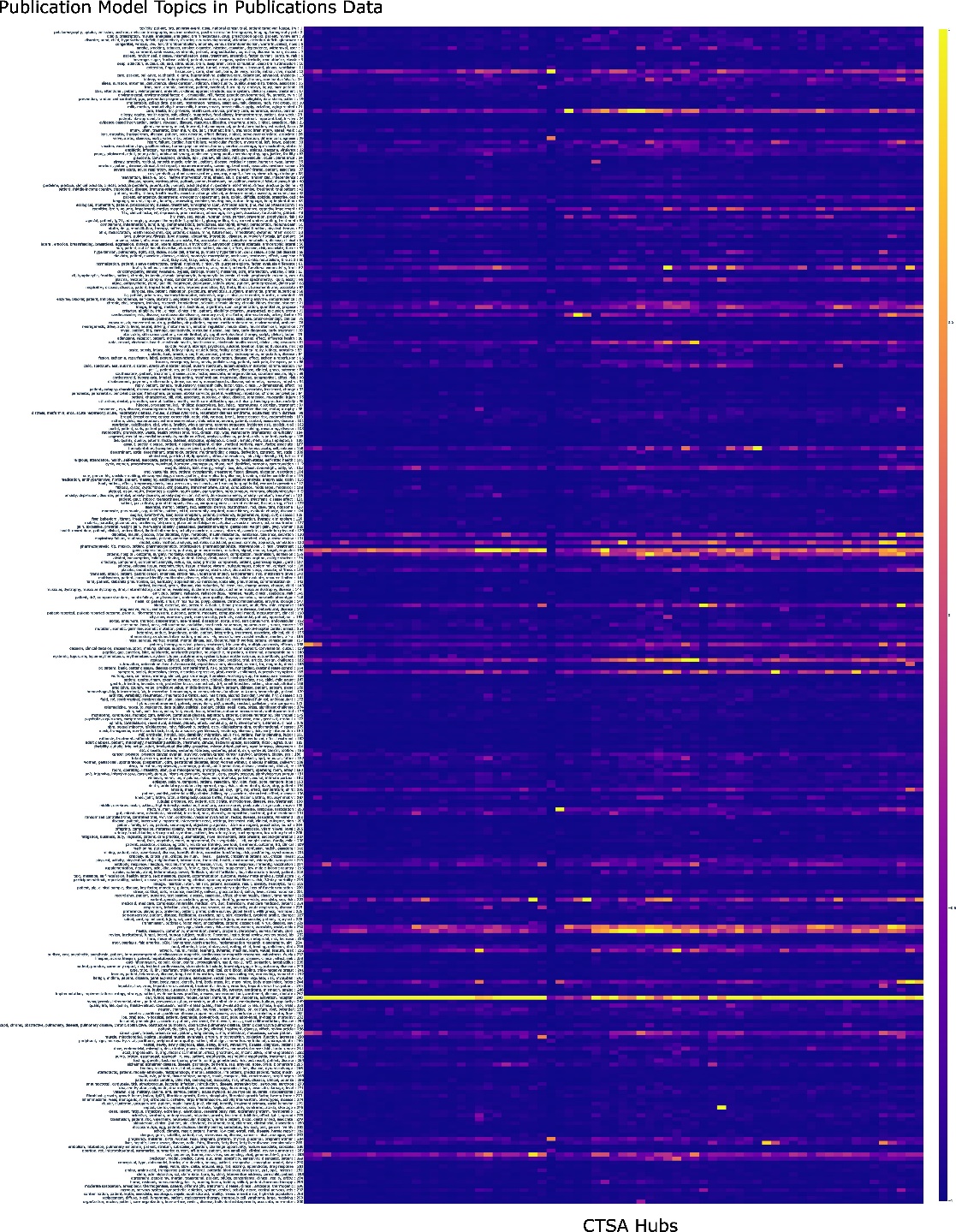 |
| --- |
| **Supplementary Figure 3**: Heatmap visualizing the distribution of Publication Model Topics on Publication Data across organizations/hubs. The topic distribution matrix from the LDA model was used to generate this heatmap. Blue indicates low topic prevalence in a hub, while yellow represents high frequency. Please refer to Supplementary_Fig3_Publication_Topics_by_Hubs.jpg for a version with higher resolution. |

| 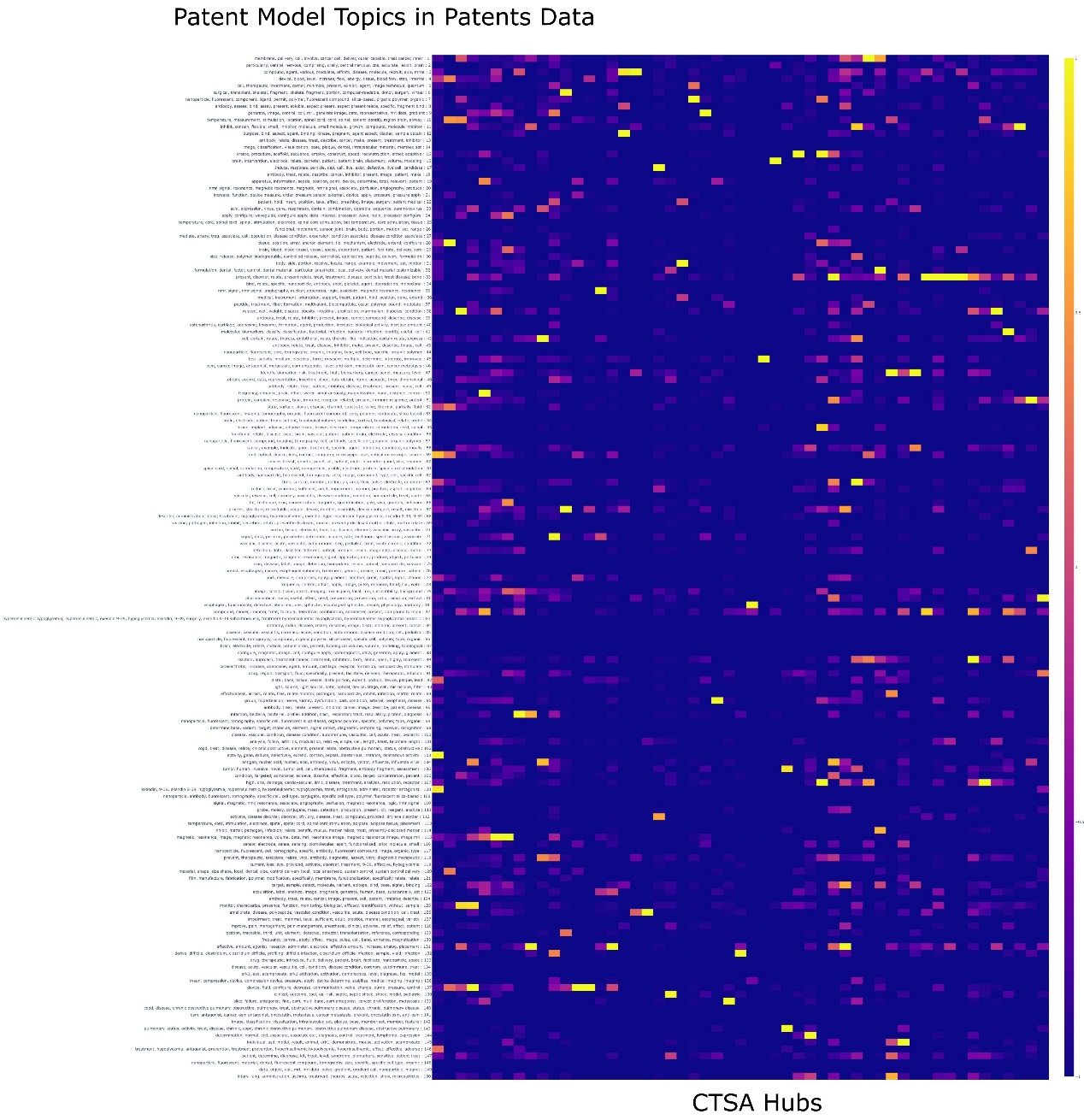 |
| --- |
| **Supplementary Figure 4**: Heatmap visualizing the distribution of Patent Model Topics on Patent Data across organizations/hubs. The topic distribution matrix from the LDA model was used to generate this heatmap. Blue indicates low topic prevalence in a hub, while yellow represents high frequency. Please refer to Supplementary_Fig4_Patent_Topics_by_Hubs.jpg for a version with higher resolution. |
| 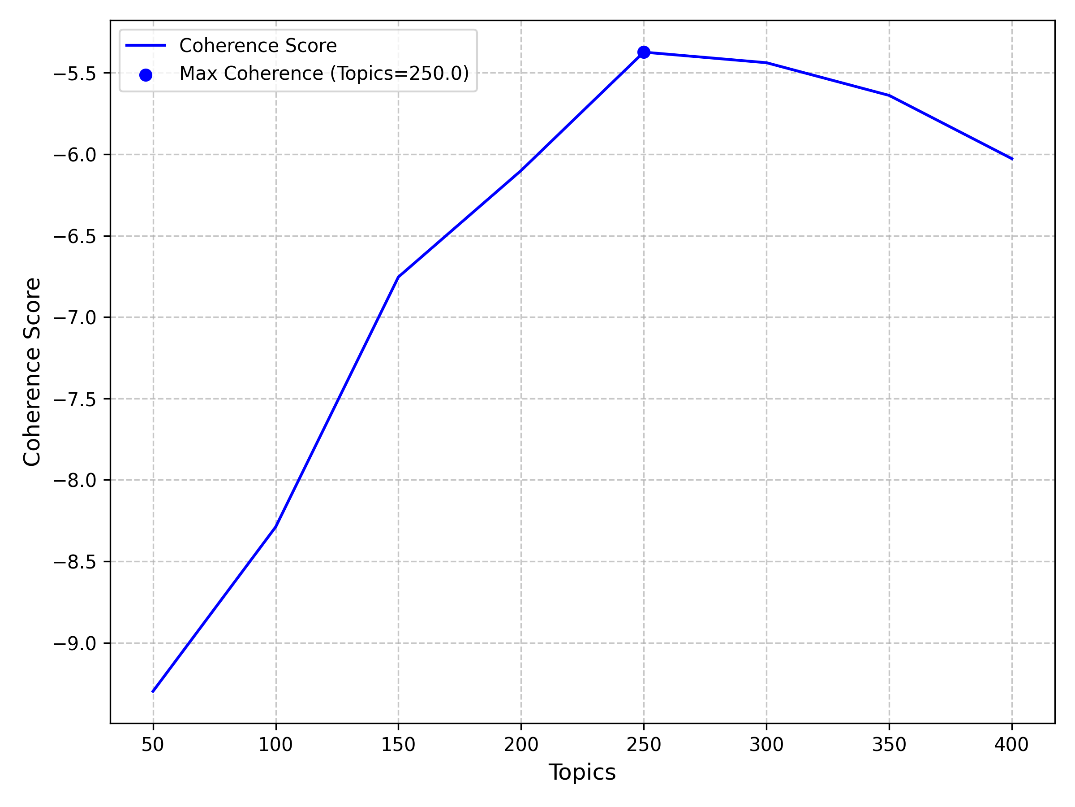 |
| **Supplementary Figure 5:** Example of testing coherence for the Publication model to identify the topic number. This number appeared to be highly sensitive to hyperparameters and was specific to the document. SME reviews were instrumental in fine-tuning the topics after the initial use of coherence, which was used to determine the topic number. |

| 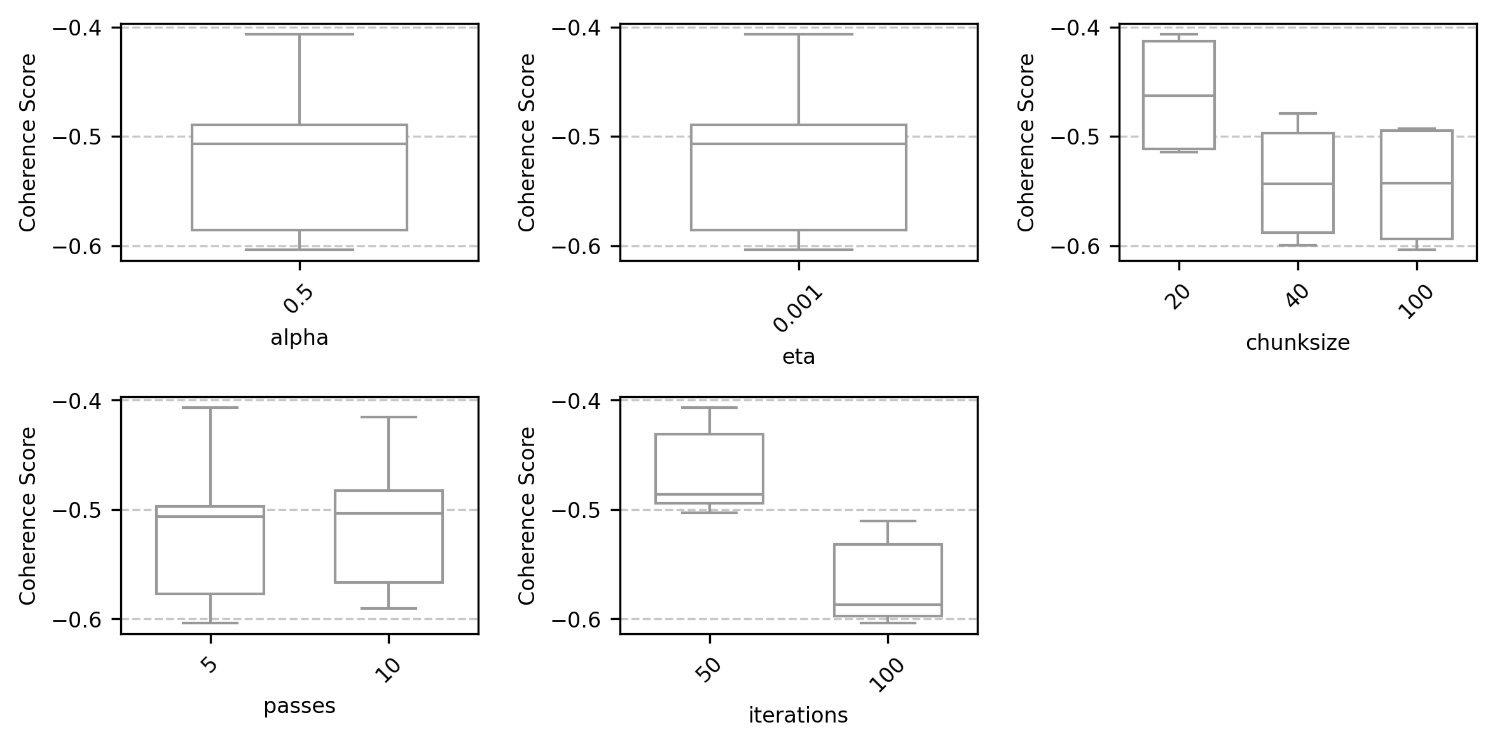 |
| --- |
| **Supplementary Figure 6**: This figure illustrates the relationship between coherence scores and various hyperparameters during the final pass of the model on Projects/Grants data. Coherence scores were used to select optimal values for chunk size, passes, and iterations, based on multiple iterations.  To further refine the model, hyperparameters such as eta and alpha were adjusted to ensure topic words accurately reflect document content. This fine-tuning aimed to optimize Latent Dirichlet Allocation (LDA), improving the coherence and interpretability of the topics.  The final tuning determined chunk size and iterations, while passes, alpha, and eta were adjusted based on topic document relationships and how the first 30 words represented the document. |

**1.2 Supplementary Tables**

**Supplemental Table 1: ChatGPT4.0 Assisted Label Prompts**

| **Prompt 1:**  Using the provided data, extract theme titles, ensuring that each theme is explicitly derived from the top 30 Relevant Words and Frequent Words in a structured, scientifically meaningful way. Relevant words are to be weighted higher than frequent words. Provide a rationale for the theme title selection, and suggest alternative refinements based on relevant and frequent words from biomedical datasets.    Every theme must contain a rigorously derived dominant theme that aligns with established scientific, clinical, economic, or policy terminology. After initial 1 by 1 completion over chat, systematically review all theme entries in table to ensure consistency and precision in theme derivation. |
| --- |
| **Input Format:**   - Relevant Words - Frequent Words |
| **Guidelines for Theme Selection:**   - Explicit & Targeted: Extract specific, high-relevance themes directly from Relevant Words and Frequent Words. - Scientifically Grounded: Use precise clinical, medical, policy, or research terminology. - Conceptually Focused: Identify clear clinical, economic, or policy-related themes with real-world relevance. - Logical: Assign themes logically structured in alignment with domain-specific knowledge. - Consistently Structured: Ensure uniform formatting. - Each theme must reflect unique relevant and frequent words, with no generic placeholders like "further specification needed." |
| **Output Format:**  Medically Informed Theme Title (Weighted by Relevant Words):  *"Concise, comprehensive title that captures the core medical theme."*  Why This Works:  ✔ "Subtheme 1" → *Explanation based on the presence of specific relevant words and how they inform the theme.*  ✔ "Subtheme 2" → *How the theme reflects clinical, research, or epidemiological aspects based on relevant words.*  ✔ "Subtheme 3" → *How the theme incorporates biomedical or patient-centered aspects.*  Alternative Refinements:  ✅ "Variation 1" (for a different emphasis within the theme, e.g., more clinical or epidemiological focus)  ✅ "Variation 2" (if shifting focus to another important subtopic related to the dataset)  ✅ "Variation 3" (if prioritizing another key biomedical aspect found in the data) |
| **Prompt 2:**  Using the provided data, conducts quality control & final review:   - Ensure Every Row is Filled: No blanks - Verify Precision: Each theme must directly reflect relevant and frequent words - Explicit Term Alignment: Confirm that every theme explicitly includes terms directly traceable to relevant and frequent words. - No Vague Labels: Keep themes specific and structured. - Scientific Precision: Ensure the terminology is precise, domain-specific, and scientifically grounded. - Reapply the Rules for Consistency: Standardized logic across all rows. |

**Supplemental Table 2: Illustrative Example of SME Tagged Topics Built on Publication Dataset Analyzed with the Publication LDA Topic Model**

| **Topic No.** | 22 |
| --- | --- |
| **ChatGPT_4.0_Assisted_Label** | Mobile Health in HIV Adherence |
| **Relevant Topic Words** | woman live hiv, health policy, apps, art adherence, care access, provider pcp, mobile health, latina woman, hiv diagnosis, neurocognitive impairment, old people, economic burden, transcribed, primary care practice, pws, interagency hiv wihs, art initiation, learner, notification, hiv treatment, treatment compare, hiv care, people live hiv, woman live, depression treatment, medicare part, virus hiv infection, hiv clinic, rosuvastatin, moral |
| **Potential Clinical Benefits** | True |
| **Example of Clinical Benefits Relevant Words** | Treatment |
| **Example of Clinical Benefits in TSBM Framework** | Clinical Benefits > Procedures & Guidelines >> Therapeutic Procedures |
| **Example of Clinical Benefit Represented in Paper** | Rice WS, Turan B, Fletcher FE, Nápoles TM, Walcott M, Batchelder A, Kempf MC, Konkle-Parker DJ, Wilson TE, Tien PC, Wingood GM, Neilands TB, Johnson MO, Weiser SD, Turan JM. A Mixed Methods Study of Anticipated and Experienced Stigma in Health Care Settings Among Women Living with HIV in the United States. AIDS Patient Care STDS. 2019 Apr;33(4):184-195. doi: 10.1089/apc.2018.0282. PMID: 30932700; PMCID: PMC6459270. |
| **LDA Score Associated with Clinical Paper Example** | 0.45 |
| **Potential Community & Public Health Benefits** | True |
| **Example of Community & Public Health Benefits Relevant Words** | Access, Apps |
| **Example of Community & Public Health Benefits in TSBM Framework** | - Community & Public Health Benefits > Health Care Characteristics >> Health Care Accessibility - Community & Public Health Benefits > Health Activities & Products >> Consumer Software |
| **Example of Community & Public Health Benefits Represented in Paper** | Masiano SP, Martin EG, Bono RS, Dahman B, Sabik LM, Belgrave FZ, Adimora AA, Kimmel AD. Suboptimal geographic accessibility to comprehensive HIV care in the US: regional and urban-rural differences. J Int AIDS Soc. 2019 May;22(5):e25286. doi: 10.1002/jia2.25286. PMID: 31111684; PMCID: PMC6527947. |
| **LDA Score Associated with Community Public Health Benefits Paper Example** | 0.36 |
| **Potential Economic Policy Benefits** | True |
| **Example of Economic Benefits Relevant Words** | Economic |
| **Example of Economic Benefits in TSBM Framework** | Economic Benefits > Financial Savings & Benefits |
| **Example of Economic Benefits Represented in Paper** | Sahu M, Bayer CJ, Roberts DA, van Rooyen H, van Heerden A, Shahmanesh M, Asiimwe S, Sausi K, Sithole N, Ying R, Rao DW, Krows ML, Shapiro AE, Baeten JM, Celum C, Revill P, Barnabas RV. Population health impact, cost-effectiveness, and affordability of community-based HIV treatment and monitoring in South Africa: A health economics modelling study. PLOS Glob Public Health. 2023 Sep 5;3(9):e0000610. doi: 10.1371/journal.pgph.0000610. PMID: 37669249; PMCID: PMC10479912. |
| **LDA Score Associated with Economic Paper Example** | 37669249 |
| **Potential Policy Benefits** | True |
| **Example of Policy Benefits Relevant Words** | Policy |
| **Example of Policy Benefits in TSBM Framework** | Policy Benefits > Policies & Legislation >> Policies |
| **Example of Policy Benefits Represented in Paper** | Mahone A, Enich M, Treitler P, Lloyd J, Crystal S. Opioid use disorder treatment and the role of New Jersey Medicaid policy changes: perspectives of office-based buprenorphine providers. Am J Drug Alcohol Abuse. 2023 Sep 3;49(5):606-617. doi: 10.1080/00952990.2023.2234075. Epub 2023 Jul 28. PMID: 37506336; PMCID: PMC10826857. |
| **LDA Score Associated with Policy Paper Example** | 0.20 |
| **No. of Publications Tagged with Topic 22** | 166 |
